# Evolutionarily conserved resistance to phagocytosis observed in melanoma cells is insensitive to upregulation of pro-phagocytic signals and to CD47 blockade

**DOI:** 10.1097/CMR.0000000000000629

**Published:** 2019-06-12

**Authors:** Katie L. Anderson, Kristin M. Snyder, Daisuke Ito, Debra C. Lins, Lauren J. Mills, Kipp Weiskopf, Nan G. Ring, Aaron M. Ring, Yoji Shimizu, Matthew F. Mescher, Irving L. Weissman, Jaime F. Modiano

**Affiliations:** a DVM/PhD dual degree program of the Comparative Molecular Biosciences Graduate Group; b Animal Cancer Care and Research Program; c Department of Veterinary Clinical Sciences, College of Veterinary Medicine, St. Paul; d Masonic Cancer Center; e Center for Immunology; Departments of f Laboratory Medicine and Pathology; g Pediatrics, School of Medicine; h Minnesota Supercomputing Institute; i Stem Cell Institute, University of Minnesota, Minneapolis, Minnesota; j Institute for Stem Cell Biology and Regenerative Medicine; k Ludwig Center for Cancer Stem Cell Research and Medicine; l Stanford Cancer Institute, Stanford University School of Medicine, Stanford, California, USA

**Keywords:** CD47, immunoevasion, macrophage, melanoma, phagocytosis

## Abstract

Therapeutic activation of macrophage phagocytosis has the ability to restrain tumour growth through phagocytic clearance of tumour cells and activation of the adaptive immune response. Our objective for this study was to evaluate the effects of modulating pro- and anti-phagocytic pathways in malignant melanoma. In order to identify evolutionarily conserved mechanisms of resistance that may be important for melanoma cell survival, we utilized a multi-species approach and examined the phagocytosis of human, mouse, and dog melanoma cells. We observed that melanoma cells from all three species displayed unexpected resistance to phagocytosis that could not be fully mitigated by blockade of the ‘don’t eat me’ signal CD47 or by chemotherapeutic enhancement of known ‘eat me’ signals. Additionally, CD47 blockade failed to promote anti-melanoma immune responses or tumour regression *in vivo*. This melanoma resistance to phagocytosis was not mediated by soluble factors, and it was unaffected by siRNA-mediated knockdown of 47 prospective ‘don’t eat me’ signals or by CRISPR-Cas-mediated CD47 knockout. Unexpectedly, CD47 knockout also did not enhance phagocytosis of lymphoma cells, but it eliminated the pro-phagocytic effect of CD47 blockade, suggesting that the pro-phagocytic effects of CD47 blockade are due in part to Fc receptor engagement. From this study, we conclude that melanoma cells possess an evolutionarily conserved resistance to macrophage phagocytosis. Further investigation will be needed to overcome the mechanisms that mediate melanoma cell resistance to innate immunity.

## Abstract 2

Supplemental Digital Content is available in the text.

## Introduction

Classically activated macrophages have the potential to mediate robust anti-tumour immunity through phagocytic clearance of tumour cells and antigen presentation to the adaptive immune system [1]. This macrophage function relies on a balance of pro-phagocytic (‘eat me’) and anti-phagocytic (‘don’t eat me’) signals expressed on the surface of target cells [2].

Pro-phagocytic signals, such as phosphatidylserine and calreticulin, are expressed on the surface of cells undergoing immunogenic cell death and can be induced by treatment with anthracycline chemotherapy drugs, such as doxorubicin [2,3].

The anti-phagocytic signal CD47 is expressed on a wide variety of cancers [4–6] and acts as an innate immune checkpoint in the tumour microenvironment [7]. CD47 binds to the SIRPα receptor on macrophages and initiates a signalling cascade within the macrophage to inhibit phagocytosis [4–6]. Therapies that block the interaction of CD47 and SIRPα have been shown to stimulate tumour cell phagocytosis *in vitro* and induce anti-tumour immune responses *in vivo* [4,5,8,9], and CD47 blockade is being tested in clinical trials [10].

Phagocytosis stimulated by CD47 blockade results in antigen presentation and activation of the adaptive immune response [8,9]. Thus, therapies to enhance phagocytosis may synergize with existing immunotherapies that seek to reactivate the adaptive immune system. Many of these immunotherapies have been pioneered for the treatment of malignant melanoma. Melanoma is the most lethal form of skin cancer due to its aggressive nature and propensity for metastasis [11]. The use of immunotherapy has revolutionized the treatment of melanoma and led to durable remissions in a number of patients. However, the fact that more than 40% of patients with malignant melanoma do not respond to immune checkpoint blockade using combination anti-CTLA-4 and anti-PD-1 therapy underscores the need to develop additional therapeutics for the treatment of this disease [12,13]. Macrophage-activating therapies have the potential to promote durable responses in the subset of patients that display resistance to current treatments.

Malignant melanoma occurs in a number of other species, including mice and dogs, which can serve as translational models for the human disease [14–17]. In addition to providing a framework for preclinical testing, studying melanoma cells from multiple species facilitates the identification of evolutionarily conserved mechanisms of immunoevasion that are likely to be important for tumour cell survival [16]. Therefore, we utilized a multi-species approach to evaluate the response of human, mouse and canine melanoma cells to modulation of phagocytic signals. We demonstrate that melanoma cells from all three species display a conserved mechanism of resistance to phagocytosis that cannot be overcome by modulation of known pro- and anti-phagocytic signals and may be related to changes in antibody-mediated effects.

## Materials and methods

Additional methods can be found in Supplemental digital content 1, http://links.lww.com/MR/A158.

### Cell lines and culture

Melanoma cell lines (human M14 and M14-GFP: Dr. David Cheresh, University of California San Diego, USA; mouse B16-OVA: Dr. Ross Kedl, University of Colorado Denver, USA [18]; canine TLM1, CMGD2, and CMGD5: obtained as described [19]), mammary cancer cell lines (human MCF7: American Type Culture Collection (ATCC), mouse 4T1: Dr. Kaylee Schwertfeger, University of Minnesota, USA; canine CMT12: Dr. Curtis Bird, Auburn University, USA; feline K12: Dr. Bill Hardy, Rockefeller University, USA [20]), osteosarcoma cell lines (human SAOS2: ATCC; mouse K12: National Cancer Institute, Bethesda, MD, USA; canine OSCA-40, OSCA-78: obtained as described [21]) were cultured in Dulbecco’s Modified Eagle Medium with 10% foetal bovine serum and 100 μg/ml Primocin. Note: Both the feline mammary cancer and mouse osteosarcoma cell lines were originally named K12. Here, the feline cell line is referred to as K12 and the mouse line as K12 murine osteosarcoma. CLBL1 canine lymphoma cells (from Dr. Barbara Rütgen, University of Vienna, Austria [22]), A20 mouse lymphoma cells (ATCC), and Raji human lymphoma cells (ATCC) were cultured as described. All cell lines used tested mycoplasma negative by PCR and were authenticated using single tandem repeat profiling through DDC Medical or Idexx Bioresearch.

### Therapeutic agents

The high-affinity SIRPα protein CV1-hIgG4 [23] and the anti-CD47 mAb Hu5F9-G4 [24] were produced as described. The corresponding isotype control, huIgG4, mouse anti-CD47 antibody (clone MIAP301), its corresponding isotype control, mIgG2a, and anti-CD271 (clone ME20.4) were obtained from eBioscience (San Diego, California, USA).

### Detection of CD47 expression and blocking of the CD47/SIRPα axis

Binding of AlexaFluor488 Hu5F9-G4, BV786 mouse anti-human CD47 (Clone B6H12; BD Biosciences, San Jose, California, USA), or PE anti-mouse CD47 (Clone MIAP301; Biolegend, San Diego, California, USA) was assessed using an LSRII flow cytometer, and geometric mean fluorescence intensity was determined using FlowJo. To analyse the blocking ability of CV1-hIgG4, 1 × 10^6^ cells were incubated with varying concentrations of CV1-hIgG4 for 15 minutes on ice. Cells were subsequently labelled using AlexaFluor488 Hu5F9-G4. Analysis was performed as described above, and data were fit to sigmoidal dose-response curves using Prism 6.

### Macrophage phagocytosis assays

We used mouse J774 cells, non-obese diabetic, severe combined immunodeficient, common gamma chain knockout mouse (NOD-SCID-Gamma, or NSG) macrophages, and human macrophages for our experiments. J774 macrophages were activated 24 hours before phagocytosis assays using 100 ng/ml recombinant mIFNγ (eBioscience). Cancer cells were either GFP+ or labelled with carboxyfluorescein succinimidyl ester (CFSE) (Thermo Fisher Scientific, Waltham, Massachusetts, USA) and were incubated with 10 μg/ml of CD47 blocking reagents, isotype controls, or tumour-targeting antibodies for 30 minutes. Macrophages were then co-cultured in non-adherent plates with cancer cells at a 1:1 (J774) or 1:2 (NSG, human macrophages) ratio. Phagocytosis was analysed by flow cytometry after 2 hours. Mouse macrophages were identified with PE/Cy7- or allophycocyanin (APC)-anti-mouse F4/80 (Biolegend), and human macrophages were identified with APC-anti-human CD206 (Biolegend). Phagocytosis was quantified as the percentage of F4/80+ or CD206+ cells that engulfed CFSE+/GFP+ tumour cells per total F4/80+ or CD206+ population. For soluble factor assays, Raji cells and J774 macrophages were suspended in supernatant harvested from cultured M14 melanoma cells titrated with new IMDM medium for the duration of the assay.

### Harvest of OT-I and OT-II T cells

OT-I.PL mice and OT-II.PL mice were provided by the Mescher laboratory and Dr. Marc Jenkins (University of Minnesota). OT-I and OT-II T cells were harvested as described [25]. OT-I/PL LN cells were enriched for CD8^+^CD44^low^ cells by negative selection using MACS magnetic cell separation (Miltenyi Biotec, Bergisch Gladbach, Germany) and were 95% CD8^+^ and 0.5% CD44^high^ post-separation [25]. OT-II T cells were purified in a similar fashion, selecting for CD4^+^CD44^low^ cells to 80% purity.

### *In vivo* T cell activation

Syngeneic CD45.2 C57BL/6 mice were injected subcutaneously (SC) with 2 × 10^5^ B16-OVA cells (Supplemental digital content 2, http://links.lww.com/MR/A159). Mice received intraperitoneal (IP) injections of MIAP301 or control mouse IgG2a (200 μg) on days 9 and 11. On day 13, OT-I and OT-II T cells were harvested and purified as described and labelled with CellTrace Violet (CTV; Thermo Fisher). As negative controls, one non-tumour bearing mouse received OT-I and OT-II cells, and one tumour-bearing mouse did not receive OT-I or OT-II cells. As a positive control, one tumour-bearing mouse received OT-I and OT-II cells along with 50 μg OVA_257–264_ peptide and 50 μg OVA_323–339_ peptide. Three days following adoptive transfer, the tumour-draining lymph node, non-draining (contralateral) lymph node, and tumour were harvested and homogenized into single cell suspensions. Cells were labelled with antibodies against CD8, CD4, CD69, CD44, Thy1.1, and CD45.1 (Biolegend). T cell activation and percent division was assessed by CTV dilution using an LSRII flow cytometer.

### Tumour control assay

Syngeneic CD45.2 C57BL/6 recipient mice were injected SC with 2 × 10^5^ B16-OVA cells. Mice received IP injections of MIAP301 or control IgG2a (200 μg) on days 13, 15, and 17. On day 17, OT-I and OT-II T cells were harvested, purified, and labelled with CTV as described. Control mice were treated as described in the T cell activation assay. Tumour growth was measured daily until tumours reached maximum size, as established by the Institutional Animal Care and Use Committee of the University of Minnesota, which was approximately 3 weeks.

### Chemotherapeutic treatment

B16-OVA cells were treated with 0.03 μM doxorubicin for 24 hours. Cells were harvested and stained with propidium iodide and APC Annexin V (Biolegend). Membrane phosphatidylserine exposure was evaluated by flow cytometry and is represented by the %Annexin V+PI- cells. Alternatively, cells were labelled with an Alexa Fluor 647 anti-mouse calreticulin antibody (clone ERP3924; Abcam, Cambridge, England) and incubated on ice for 30 minutes before analysis.

### Brefeldin A treatment

M14-GFP or Raji cells labelled with CFSE were incubated with 5.0 μg/ml Brefeldin A (BFA) solution for 2 hours before placement in phagocytosis assays. 5.0 μg/ml BFA solution was added to the assay medium.

### Statistical analysis

Phagocytosis assays were analysed by Mann–Whitney *U* test (comparing the experimental condition to the no antibody control) or by 1-way analysis of variance. T cell activation and tumour growth were analysed by Mann–Whitney *U* test (comparing the experimental condition to the no antibody or tumour only controls). Statistical analysis was performed using Prism (GraphPad).

## Results

### Mouse melanoma cells display resistance to phagocytosis that is not fully mitigated by CD47 blockade

**Fig. 1:**
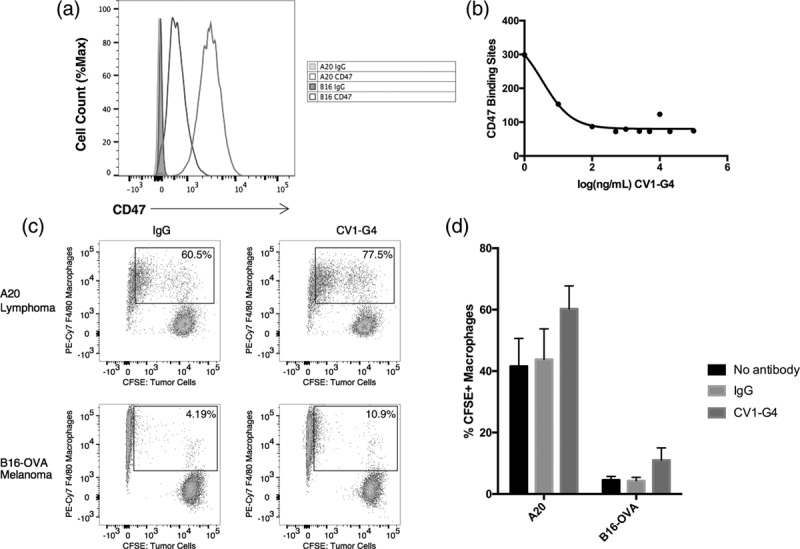
Mouse melanoma displays resistance to phagocytosis that is not mitigated by CD47 blockade. (a) CD47 expression: labelling of mouse melanoma (B16-OVA) and lymphoma (A20) cells with the anti-CD47 mAb MIAP301 (PE) as compared to an isotype control was detected by flow cytometry. (b) Efficacy of CD47 blockade: cells were incubated with CV1-G4 at the indicated concentrations for 15 minutes, followed by labelling with MIAP301 (PE) to detect unoccupied CD47 binding sites. (c) Phagocytosis assay: CFSE-labelled tumour cells were incubated with J774 macrophages in the presence of control IgG4 or CV1-G4. Phagocytosis was quantified as the percent of F4/80^+^ J774 cells that engulfed CFSE^+^ tumour cells per total F4/80^+^ population. Flow cytometry plots represent the mean percent phagocytosis. (d) Quantification of phagocytosis assays: The data are a summary of six experiments (B16-OVA) or three experiments (A20) repeated in triplicate (mean ± SEM).

We began to evaluate the role of CD47 in melanoma cell phagocytosis by measuring CD47 expression on mouse B16 melanoma cells. Mouse A20 lymphoma cells, which are sensitive to CD47 blockade [6,8], were used as a positive control. We confirmed the surface expression of CD47 in both cell types (Fig. 1a). To assess the ability of a SIRPα-Fc fusion protein, CV1-hIgG4 [23], to block CD47, we incubated B16 and A20 cells with increasing concentrations of unlabelled CV1-hIgG4, followed by staining with a fluorescently-conjugated anti-CD47 antibody (MIAP301). CV1-hIgG4 blocked interactions between MIAP301 and mouse CD47 on the surface of both cell types (Fig. 1b, Supplemental digital content 3A, http://links.lww.com/MR/A160). To evaluate the ability of CD47 blockade to enhance phagocytosis of mouse melanoma cells *in vitro*, we incubated CFSE-labelled B16 cells with activated mouse J774 macrophages in the presence or absence of CV1-hIgG4. A20 cells (positive control) displayed a high basal level of phagocytosis (41.6 ± 9%), which was increased upon CD47 blockade (60.3 ± 7.5%; Fig. 1c and d). In contrast, B16 cells were poorly phagocytosed (4.6 ± 1.2%; Fig. 1c and d). Treatment with CV1-hIgG4 lead to a modest increase in melanoma cell phagocytosis (10.6 ± 3.9%); however, the overall level of melanoma cell phagocytosis remained low compared to lymphoma cells (Fig. 1c and d). We observed similar results using primary mouse bone marrow-derived macrophages, indicating that the defect in phagocytosis was not macrophage-dependent (data not shown).

### T cell activation following CD47 blockade does not enhance rejection of B16-OVA melanoma tumours

**Fig. 2:**
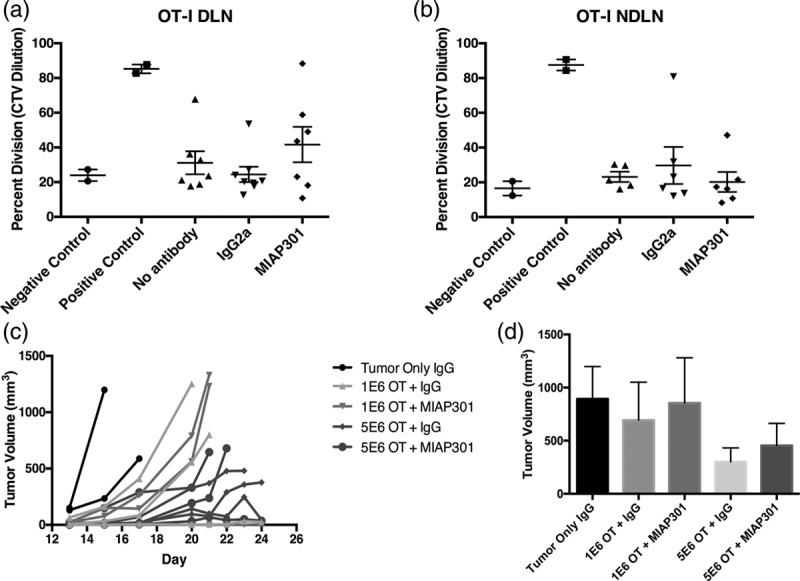
T cell activation following CD47 blockade does not enhance rejection of B16-OVA melanoma tumours. (a and b) B16-OVA tumour-bearing mice received injections of PBS (no antibody), IgG2a isotype control, or anti-CD47 mAb (MIAP301) on days 10 and 12 (200 μg IP/injection or equivalent volume of PBS). 1 × 10^6^ OT-I T cells were purified, labelled with CellTrace Violet (CTV), and injected IV on day 14. Negative control: OT-I and OT-II T cells were injected into a non-tumour bearing mouse. Positive control: OT-I and OT-II T cells were injected into a tumour bearing mouse with SIINFEKL and OVA_323-339_ peptides. On day 17, the tumour-draining lymph node (DLN) and the contralateral, non-draining lymph node (NDLN) were harvested, and cells were analysed by flow cytometry. Gating of OT-I cells was performed using congenic markers and percent division was determined by CTV dye dilution. Each symbol represents a different mouse; assay was performed three times with three mice per experimental group and 1–2 mice per control group. (a) Division of OT-I cells in the DLN. (b) Division of OT-I cells in the NDLN. (c and d) B16-OVA tumour-bearing mice received IP injections of either IgG2a or MIAP301 on days 13, 15, and 17 (200 μg IP/injection). On day 17, OT-I and OT-II T cells were purified, labelled with CellTrace Violet (CTV), and injected intravenously. Groups received either 1.0 × 10^6^ or 5.0 × 10^6^ of each OT-I and OT-II cells. Tumour growth was measured with callipers, and volume was calculated as length × width^2^ × 0.52. (c) Graph represents a spider plot of tumour growth in individual mice. (d) Bar graph (mean ± SEM) representing tumour burden at the experimental endpoint (day 24 or maximum tumour volume). IP, intraperitoneal.

Phagocytosis in response to CD47 blockade elicits an anti-tumour adaptive immune response in the syngeneic A20 model [8,9]. To assess antigen-specific T cell responses against melanoma *in vivo*, mice engrafted with B16-OVA tumours received two injections of PBS, anti-CD47 antibody (MIAP301), or isotype control antibody (Supplemental digital content 2, http://links.lww.com/MR/A159). Transgenic OVA-specific CD8^+^ (OT-I) and CD4^+^ (OT-II) T cells labelled with CTV were adoptively transferred into mice 2 days later and harvested for analysis after an additional 3 days [26,27]. MIAP301 lead to an increase in the average percent division of OT-I cells (41.7 ± 10.2%) in the tumour-draining lymph node (DLN) as compared to the isotype control (24.5 ± 4.4%) (Fig. 2a). OT-I cells harvested from the non-draining (contralateral) lymph node (NDLN) showed no change, indicating that a tumour-specific response had occurred (Fig. 2b). There was no difference in cell division of OT-II T cells in the DLN and the NDLN (data not shown).

To determine if this observed increase in antigen-specific CD8^+^ T cell activation could mediate tumour rejection, mice bearing B16-OVA tumours were treated with three injections of MIAP301 or isotype control, and both OT-I cells and OT-II cells were adoptively transferred following antibody treatment. As expected, transferring higher numbers of OT-I and OT-II cells provided greater tumour control (Fig. 2c and d). However, we observed no difference in tumour growth between the isotype control- and MIAP301-treated animals over the course of 3 weeks (Fig. 2c and d).

### Melanoma cell resistance to phagocytosis is evolutionarily conserved

**Fig. 3:**
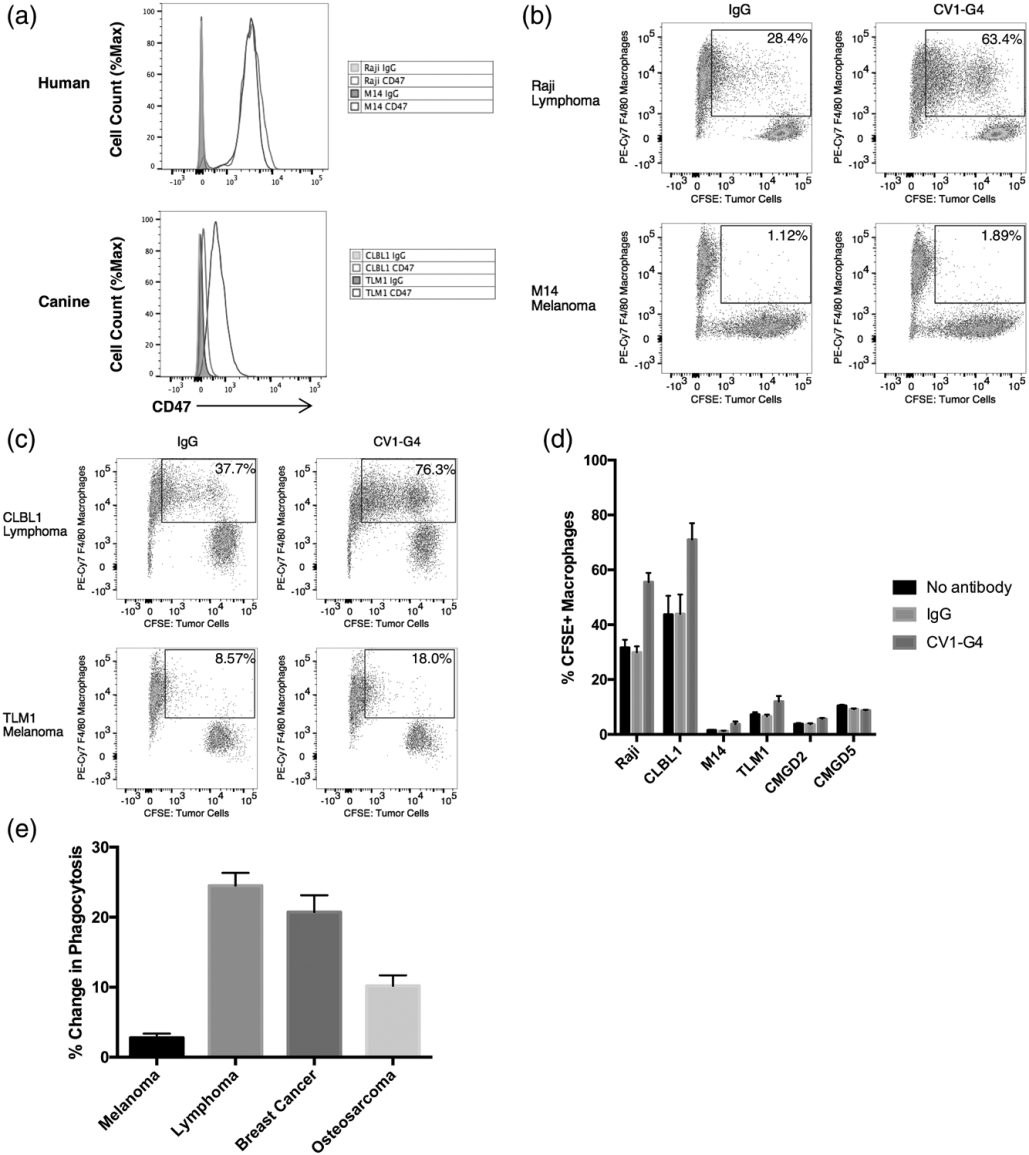
Melanoma cell resistance to phagocytosis is conserved among different species. (a) CD47 expression: labelling of human melanoma (M14) and lymphoma (Raji) cells and canine melanoma (TLM1) and lymphoma (CLBL1) cells with the anti-CD47 mAb 5F9 (Alexa 488) as compared to an isotype control was detected by flow cytometry. (b) Phagocytosis of human tumour cells: CFSE-labelled tumour cells were incubated with J774 macrophages in the presence of control IgG4 or CV1-G4. Phagocytosis was quantified as the percent of F4/80^+^ J774 cells that engulfed CFSE^+^ tumour cells per total F4/80^+^ population. Flow cytometry plots represent the mean percent phagocytosis. (c) Phagocytosis of canine tumour cells as described in (b). (d) Quantification of phagocytosis assays: The data are a summary of three experiments (CLBL1, TLM1, CMGD2, CMGD5), 12 experiments (M14), or 16 experiments (Raji) repeated in triplicate (mean ± SEM). (e) Summary of phagocytosis assays for all species tested.

To evaluate whether melanoma cell resistance to phagocytosis was unique to mouse cells, we examined the sensitivity of human and canine melanoma cells to phagocytosis. We confirmed that human (M14) and canine (TLM1) melanoma cell lines expressed CD47 at levels comparable to human (Raji) and canine (CLBL1) lymphoma cells (Fig. 3a). In a competition assay, CV1-hIgG4 efficiently blocked interactions between anti-CD47 antibodies and CD47 expressed on human and canine melanoma cells (Supplemental digital content 3B and 3C, http://links.lww.com/MR/A160). Raji and CLBL1 lymphoma cells demonstrated a sensitivity to phagocytosis (31.6 ± 2.9% and 50.3 ± 6.5%, respectively) that was enhanced by the addition of CV1-hIgG4 (55.6 ± 3.3% and 80.0 ± 2.3%, respectively, Fig. 3b–d). While we observed small increases in M14 and TLM1 melanoma cell phagocytosis in response to CD47 blockade, the overall percent phagocytosis of these cells (3.7 ± 1.0% and 12.4 ± 2.0%, respectively) remained low as compared to lymphoma cells (Fig. 3b–d). Similarly, low levels of phagocytosis were observed using two additional canine melanoma cell lines, CMGD2 and CMGD5 (Fig. 3D), and two additional human melanoma cell lines, SKMEL3 and SKMEL28 (Supplemental digital content 4, http://links.lww.com/MR/A161).

To compare the phagocytosis of other solid tumour cells to melanoma cells, we performed assays using human, canine, feline, and mouse breast/mammary cancer cells and osteosarcoma cells. Breast/mammary cancer and osteosarcoma cells displayed lower basal levels of phagocytosis (10.5 ± 1.0% and 15.6 ± 1.3%, respectively) than lymphoma cells, but remained more sensitive to phagocytosis than melanoma cells (Supplemental digital content 5A and 5B, http://links.lww.com/MR/A162). In contrast to melanoma cells, both cell types exhibited a substantial increase in phagocytosis (31.2 ± 2.6% and 25.8 ± 2.1%, respectively) in response to CD47 blockade (Fig. 3e).

### Modulation of pro-phagocytic signals fails to enhance phagocytosis

**Fig. 4:**
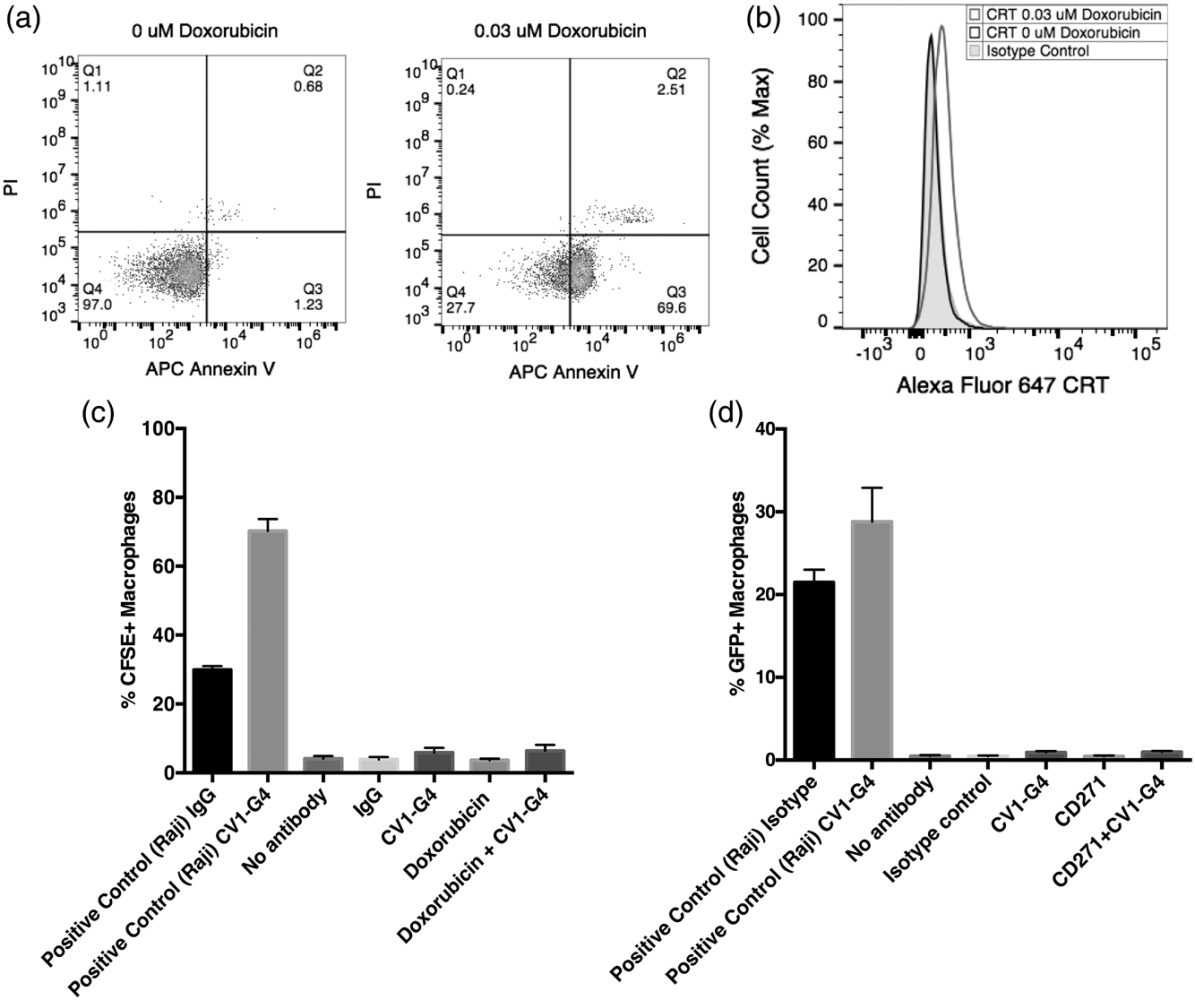
Modulation of pro-phagocytic signals fails to enhance phagocytosis. B16-OVA cells were treated with the indicated doses of doxorubicin for 24 hours. (a) Phosphatidylserine (PS) exposure following doxorubicin treatment: display of membrane PS was evaluated by flow cytometry and is represented by the %Annexin V+Propidium Iodide- cells. (b) Calreticulin (CRT) exposure following doxorubicin treatment: cells were labelled with an anti-mouse CRT antibody (Alexa Fluor 647) and analysed by flow cytometry. (c) Phagocytosis assay: GFP^+^ B16-OVA tumour cells were treated for 24 hours with 0.03 μM doxorubicin chemotherapy or PBS. Treated cells were incubated with J774 macrophages in the presence of control IgG4 or CV1-G4. Phagocytosis was quantified as the percent of F4/80^+^ J774 cells that engulfed CFSE^+^ tumour cells per total F4/80^+^ population. Summary of three experiments performed in triplicate (mean ± SEM). (d) M14-GFP tumour cells were incubated with J774 macrophages in the presence of control IgG4, CV1-G4, anti-CD271, or a combination of antibodies. Phagocytosis was quantified as described in (a). Two experiments were performed in triplicate (mean ± SEM).

To test the effects of pro-phagocytic signal expression on phagocytosis, we exposed B16 melanoma cells to doxorubicin (0.03 μM) for 24 hours and demonstrated induction of the pro-phagocytic signals calreticulin and phosphatidylserine (PS) (Fig. 4a and b). We then placed doxorubicin-treated B16 cells in a phagocytosis assay in the presence or absence of CV1-hIgG4. We observed no difference in phagocytosis following doxorubicin treatment alone or in combination with CD47 blockade (Fig. 4c).

Combining a tumour cell-specific mAb with CD47 blockade has been shown to enhance phagocytosis by providing a pro-phagocytic stimulus through the Fc receptor [23,28]. Thus, we incubated M14 cells with an antibody targeting the melanoma antigen CD271 and incubated SKMEL3 and SKMEL28 cells with an antibody targeting the melanoma antigens GD2/3 [29]. Combination therapy failed to enhance phagocytosis of M14 cells (Fig. 4d) or SKMEL3 cells (Supplemental digital content 4A and 4B, http://links.lww.com/MR/A161). CD47 blockade did not promote phagocytosis of SKMEL28 cells, although combination CD47 blockade with an anti-GD2/3 opsonizing antibody showed modest enhancement of phagocytosis using NSG macrophage effectors (1.04 ± 0.06% (no antibody), 1.97 ± 0.05% (CV1), and 22.5 ± 0.76% (CV1 + anti-GD2/3). Curiously, this enhancement was not apparent using human macrophage effectors (Supplemental digital content 4A and 4B, http://links.lww.com/MR/A161).

### Secretion of soluble ‘don’t eat me’ signals is not responsible for melanoma cell resistance to phagocytosis

**Fig. 5:**
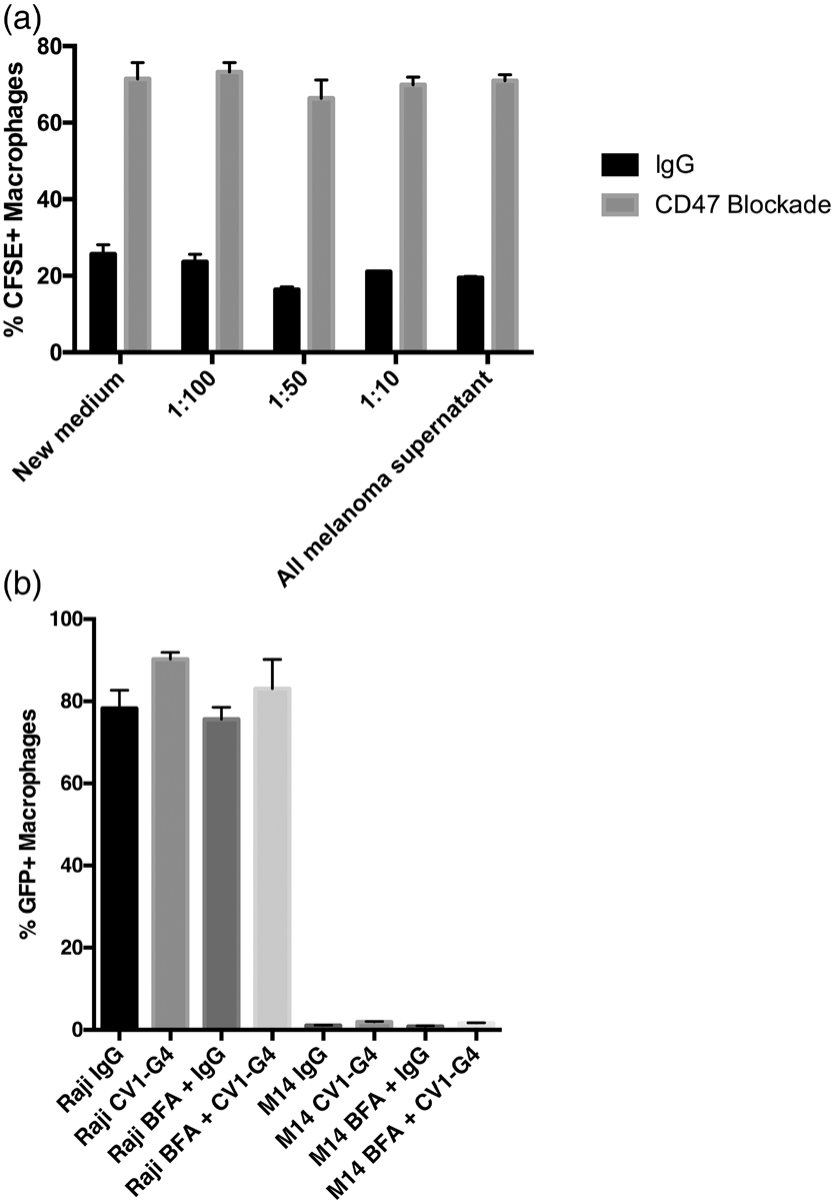
Secretion of soluble ‘don’t eat me’ signals is not responsible for melanoma resistance to phagocytosis. (a) Effects of melanoma supernatant on lymphoma cell phagocytosis: CFSE-labelled human lymphoma (Raji) cells were incubated with J774 macrophages in the presence of control IgG4 or CV1-G4 while suspended in supernatant harvested from cultured M14 melanoma cells titrated with new medium. Phagocytosis was quantified as the percent of F4/80^+^ J774 cells that engulfed CFSE^+^ tumour cells per total F4/80^+^ population. The data show one representative experiment of three experiments repeated in triplicate done with comparable results (mean ± SEM). (b) Effect of Brefeldin A (BFA) treatment on phagocytosis: M14-GFP or CFSE+ Raji cells were pre-treated with 5.0 μg/ml BFA for 2 hours before co-culture with J774 macrophages in the presence of control IgG4 or CV1-G4. Phagocytosis was analysed by flow cytometry as in Fig. 4a. Summary of two experiments repeated in triplicate (mean ± SEM).

To test if melanoma cell resistance to phagocytosis was mediated by secreted anti-phagocytic factors, we incubated Raji cells in varying concentrations of M14 cell supernatant. We observed no defect in lymphoma cell phagocytosis or interference with CD47 blockade due to the melanoma cell supernatant (Fig. 5a). In addition, when placed in a phagocytosis assay containing both Raji and M14 cells, macrophages preferentially phagocytosed lymphoma cells (data not shown).

To address the possibility that melanoma cells secreted a locally acting or short-lived anti-phagocytic factor, we treated M14 cells with a secretory inhibitor, BFA. BFA did not prevent phagocytosis of Raji cells (Fig. 5b), although in these experiments, Raji cell phagocytosis was close to the limit of detection, so the enhancement of phagocytosis with CD47 blockade was modest. Nonetheless, melanoma cell phagocytosis was not enhanced by BFA (Fig. 5b).

### Melanoma resistance to phagocytosis cannot be overcome by knockdown of membrane-bound proteins with the potential to deliver ‘don’t eat me’ signals

To investigate the role of membrane-bound ‘don’t eat me’ signals in melanoma cell phagocytosis, we utilized data from The Cancer Genome Atlas to identify unique, highly expressed genes encoding melanoma membrane proteins (Supplemental digital content 6, http://links.lww.com/MR/A163). We also performed a literature search for immunomodulatory melanoma cell-surface proteins and for proteins that contribute to melanoma growth and metastasis. With this information, we developed a siRNA panel targeting 48 membrane-bound proteins (Supplemental digital content 7A, http://links.lww.com/MR/A164).

We optimized transfection of M14-GFP cells using a reporter siRNA (Supplemental digital content 8A, http://links.lww.com/MR/A165) and verified that transfection of a CD47-targeting siRNA reduced CD47 expression by 40–60% (Supplemental digital content 8B, http://links.lww.com/MR/A165). Seven additional genes were selected at random to evaluate the effect of siRNAs by quantitative real-time PCR. The results showed a 75–90% reduction in mRNA expression following siRNA knockdown (Supplemental digital content 8C, http://links.lww.com/MR/A165).

M14-GFP melanoma cells were transfected with randomly generated pools of four siRNAs (Supplemental digital content 7B, http://links.lww.com/MR/A164) or non-targeting control siRNA. Forty-eight hours later, we evaluated phagocytosis with or without CD47 blockade. We tested the pools of siRNAs in seven groups, each with its own set of controls. Phagocytosis of Raji lymphoma cells was a positive control. Poor macrophage viability (98% cell death) interfered with our ability to quantify phagocytosis in the second group, so these data were excluded from our analysis. Five of 54 pools slightly increased melanoma cell phagocytosis (Supplemental digital content 7C, http://links.lww.com/MR/A164). However, upon replication of these conditions, the siRNA pools did not enhance melanoma cell phagocytosis (Supplemental digital content 8D, http://links.lww.com/MR/A165).

### CD47 knockout fails to enhance phagocytosis of melanoma or lymphoma cells

**Fig. 6:**
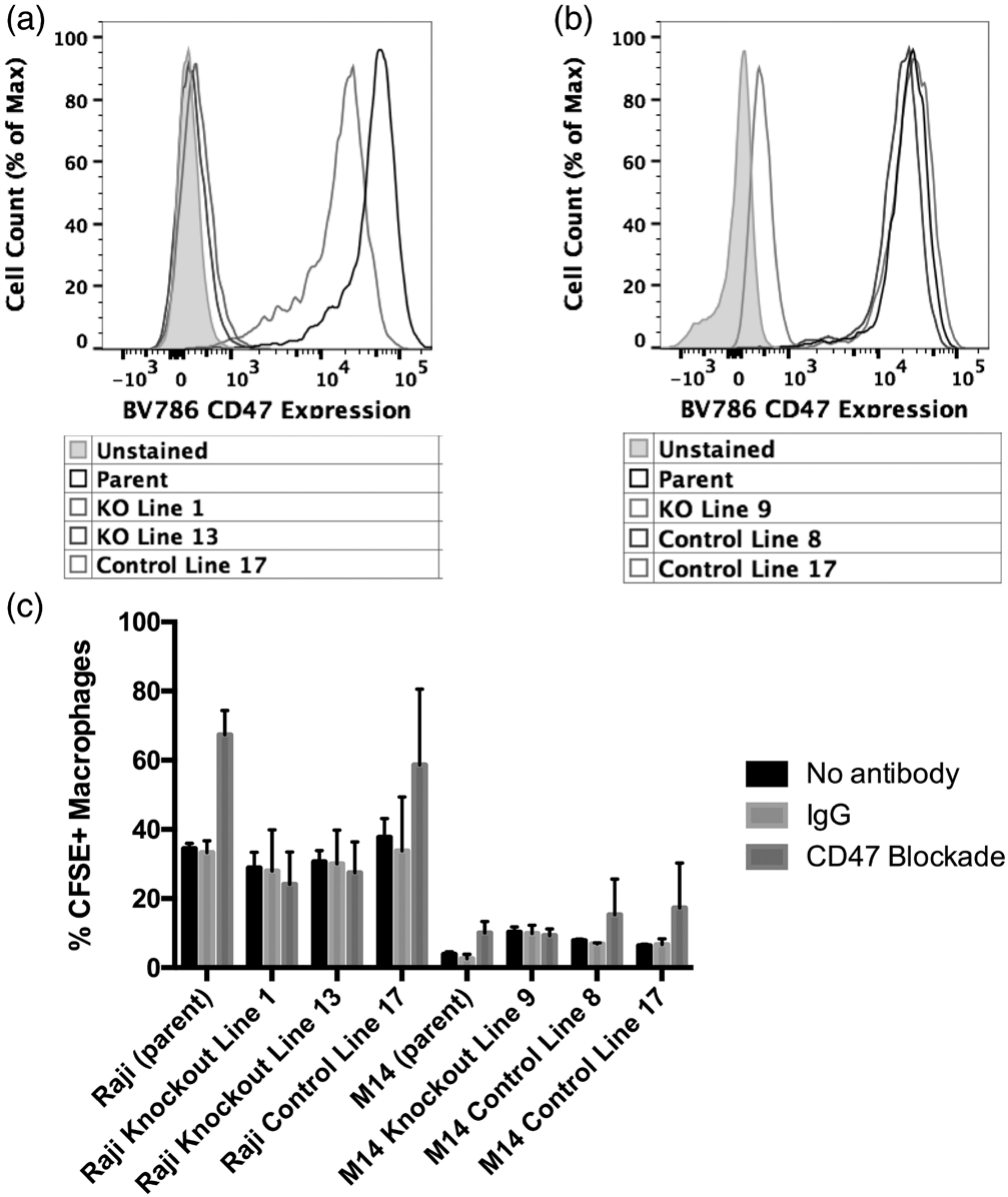
*CD47* gene knockout fails to enhance phagocytosis. Human lymphoma (Raji) and melanoma (M14) cells were transfected with CRISPR/Cas9 and a sgRNA plasmid targeting CD47. Clonal expansion and gene sequencing were performed to identify CD47 knockout clones as well as CRISPR control clones which still expressed CD47. Labelling of Raji lymphoma (a) and M14 melanoma (b) cells with an anti-CD47 mAb (BV786) was detected by flow cytometry. (c) CFSE-labelled tumour cells were incubated with J774 macrophages in the presence of no antibody, control IgG4, or CV1-G4. Phagocytosis was quantified as the percent of F4/80^+^ J774 cells that engulfed CFSE^+^ tumour cells per total F4/80^+^ population. Summary of two experiments repeated in triplicate (mean ± SEM).

While siRNA mediated knockdown reduced CD47 expression by 40–60%, it was possible that the remaining CD47 protein was capable of inhibiting phagocytosis. Therefore, we utilized the CRISPR/Cas9 system to knockout *CD47* gene expression in Raji and M14 cells. Cells that went through the same experimental procedure but retained CD47 expression were used as controls (Fig. 6a and b). M14 cells remained resistant to phagocytosis despite a lack of CD47 expression: the knockout line was phagocytosed at a rate of 10.3 ± 1.5%, while the control line was phagocytosed at a rate of 7.9 ± 0.4% (Fig. 6c).

Curiously, phagocytosis of CD47 knockout Raji lymphoma cells was similar to the untreated control cells (30.6 ± 3.3% and 37.7 ± 5.4%, respectively) (Fig. 6c). As expected, the knockout cells lost responsiveness to CD47 blockade (Fig. 6c) and restoration of CD47 expression re-established sensitivity to CD47 blockade (Supplemental digital content 9A and 9B, http://links.lww.com/MR/A166).

Both CD47 blockade and the addition of anti-CD20 as an opsonizing antibody enhanced phagocytosis of control Raji cells (from 21.3 ± 1.6% to 40.3 ± 0.4% and 33.8 ± 0.3%; Supplemental digital content 9C, http://links.lww.com/MR/A166). Although CD47 knockout Raji cells did not respond to CD47 blockade, incubation with anti-CD20 did increase phagocytosis of these cells (from 16.8 ± 0.4% to 31.2 ± 0.1%; Supplemental digital content 9C, http://links.lww.com/MR/A166).

### Proteomics analysis does not reveal differences in cell signalling following CD47 antibody blockade

To test whether inward signalling induced by anti-CD47 might be needed to sensitize the cells for phagocytosis, we completed proteomics analysis in untreated Raji and M14 parental cells, CD47 knockout cells, and CRISPR control cells, or in cells treated with isotype control, or CD47 blocking antibody (5F9-G4) for two hours. The cell signalling proteomes of CD47 knockout cells and parental cells were virtually indistinguishable (Supplemental digital content 10, http://links.lww.com/MR/A167).

## Discussion

Modulation of pro- and anti-phagocytic signals can result in tumour cell death through macrophage phagocytosis and subsequent antigen presentation to the adaptive immune system (reviewed in Ref. 10). Here, we demonstrate that melanoma cells display an evolutionarily conserved mechanism of resistance to phagocytosis that cannot be overcome by modulation of known pro- and anti-phagocytic signals and may be related to changes in antibody-mediated effects, such as interactions with the macrophage Fc receptor.

Previous studies have produced contradictory results regarding the efficacy of CD47 blockade in the treatment of melanoma. In agreement with our study, Sockolosky *et al.* [30] examined the effects of CD47 blockade in B16 mouse melanoma and demonstrated no increase in phagocytosis and no inhibition of tumour growth following treatment with a CD47 nanobody. Barkal *et al.* [31] also found no difference in the growth of CD47 knockout B16-F10 tumours compared to wild-type. However, Ngo *et al*. [29] demonstrated an increase in melanoma cell phagocytosis *in vitro* as well as a decrease in the growth and metastasis of primary human melanoma xenografts following CD47 blockade. Our work provides a systematic evaluation of melanoma cell phagocytosis by examining the effects of CD47 blockade on melanoma cells derived from human, mouse, and dog cells. Similar to the study by Ngo, we observed a small increase in melanoma cell phagocytosis *in vitro*. However, our work revealed that the overall levels of melanoma cell phagocytosis remained very low compared to the phagocytosis of lymphoma, breast/mammary gland cancer, and osteosarcoma cells. CD47 expression was found to be variable among the cell lines tested, suggesting that the difference in engulfment between melanoma and other cancer cells is not attributable to the magnitude of CD47 expression.

CD47 blockade led to a slight increase in antigen-specific CD8^+^ T cell activation *in vivo*, but this was not therapeutically relevant, as CD47 blockade failed to mediate B16 melanoma tumour regression. The differing results from our work and Sockolosky’s [30] with Ngo’s [29] could be explained by the features of the model systems: Ngo used primary human melanoma xenografts in immunodeficient mice, whereas we used a syngeneic mouse melanoma. In xenografts, only tumour cells expressing human CD47 will bind human CD47-targeting reagents, potentially achieving higher concentrations of antibody in the tumour than in syngeneic models where both malignant and normal cells can bind CD47, creating an “antigen sink” [24,30].

Enhancement of pro-phagocytic signals through treatment with chemotherapy or tumour-specific antibodies can also increase tumour cell phagocytosis [3,6,29]. We demonstrated that low-dose doxorubicin chemotherapy upregulated expression of the pro-phagocytic signals calreticulin and phosphatidylserine, but failed to increase phagocytosis of melanoma cells. In addition, the combination of a tumour-specific antibody with CD47 blockade failed to meaningfully enhance the phagocytosis of three human melanoma cell lines, although SKMEL28 cells were sensitive to phagocytosis with an opsonizing antibody. These results reflect the heterogeneity of melanoma cells, and suggest that antibody blockade might only benefit a small subset of melanoma patients.

We demonstrated that melanoma resistance to phagocytosis was not mediated by a soluble or membrane-bound ‘don’t eat me’ signal, although it remains possible that one or more ‘don’t eat me signals’ not included in our panel could mediate resistance. Genome editing experiments confirmed that loss of CD47 created only modest increases in melanoma phagocytosis. Surprisingly, elimination of CD47 did not enhance the phagocytosis of lymphoma cells, although phagocytosis of these cells was still enhanced by other opsonizing antibodies as well as by restoration of CD47 expression. This suggests that, despite the creation of CD47 blocking reagents on a human IgG4 platform [32], binding of macrophage Fc receptors still contributed to phagocytosis in this system. These antibodies may provide a ‘bridge’ between macrophages and tumour cells, triggering contacts that promote phagocytosis [33]. Proteomics analysis did not reveal any changes in inward signalling capable of sensitizing lymphoma cells to phagocytosis following CD47 blockade, although it remains possible that antibody binding induced more rapid changes that returned to baseline before analysis.

In summary, our data demonstrate that melanoma cells display an evolutionarily conserved resistance to phagocytosis that cannot be overcome by modulation of known pro- and anti-phagocytic signals. How melanoma cells evade antibody-mediated phagocytosis remains unclear, although our data show that CD47 blocking antibodies effectively bind to and block CD47 on the surface of melanoma cells and that resistance is not mediated by melanoma secreted factors, by the inability to upregulate ‘eat-me’ signals, or by the expression of a large array of ‘don’t eat me’ signals.

Evasion of phagocytosis may be an important component of the resistance to immunotherapy seen in many melanoma patients. Thus, advancing our understanding of how melanoma cells avoid elimination by the innate immune system could lead to the development of new therapeutic strategies. The use of comparative animal models has the potential to inform our understanding of conserved mechanisms of resistance that are important for melanoma cell survival and to assist in the preclinical development of efficacious therapies for advanced melanoma. Further investigation will be needed to identify the mechanisms that mediate the resistance of melanoma cells to phagocytosis.

## Acknowledgements

We thank the members of the Modiano and Weissman laboratories, especially Jens-Peter Volkmer, for technical assistance, scientific discussion, and reagents. We would also like to thank the University of Minnesota Gene Editing Shared Resource at the Masonic Cancer Center for their technical assistance.

The research reported in this manuscript was supported by grants F30 CA195973 (K.L.A.), Howard Hughes Medical Institute-Burroughs Welcome Fund Medical Research Fellowship (K.L.A.), P01 AI035296 (M.F.M.), P01 CA139490 (I.L.W.), Grant-In-Aid #22906 from the University of Minnesota Office of the Vice President for Research (J.F.M. and M.F.M.), P30 CA077598 (Comprehensive Cancer Center Support Grant to the Masonic Cancer Center, University of Minnesota, Genome Engineering Shared Resource), NCI CA16672 (RPPA Core Facility at the MD Anderson Cancer Center, University of Texas), by the GREYlong Foundation, by the Randy Shaver Cancer Research and Community Fund, by the Skippy Frank Fund for Life Sciences and Translational Research (I.L.W. and J.F.M.). J.F.M. is supported by the Alvin and June Perlman Chair in Animal Oncology. The authors gratefully acknowledge support from donors to the Animal Cancer Care and Research Program of the University of Minnesota that helped support this project.

K.L.A., D.C.L., D.I., K.W., Y.S., M.F.M., I.L.W., and J.F.M. conceived the project and assisted in experimental design. K.L.A., K.M.S., D.C.L., and N.G.R. designed and performed the original experiments. K.L.A., N.G.R, and A.M.R. performed data analysis with inputs from D.I., K.W., Y.S., M.F.M., I.L.W., and J.F.M. L.J.M. performed bioinformatics analysis and assisted in figure design. K.L.A. and J.F.M. wrote the manuscript. All authors contributed equally to manuscript revision before publication.

## Conflicts of interest

N.G.R. and A.M.R. are shareholders in Forty-Seven, Inc, and A.M.R. is a shareholder in Alexo Therapeutics. K.W. is an inventor of US patent applications pertaining to CD47-blocking assigned to Stanford University. K.W. declares consulting and/or equity ownership in Alexo Therapeutics, Inc. and Forty-Seven, Inc. I.L.W. is the co-founder and Director of Forty-Seven, Inc; he has equity and is a funded consultant for this company. There are no conflicts of interest for the remaining authors.

## Supplementary Material



## References

[1] Goswami KK Ghosh T Ghosh S Sarkar M Bose A Baral R Tumor promoting role of anti-tumor macrophages in tumor microenvironment. Cell Immunol. 2017; 316:1–10 28433198 10.1016/j.cellimm.2017.04.005

[2] Chao MP Majeti R Weissman IL Programmed cell removal: a new obstacle in the road to developing cancer. Nat Rev Cancer. 2011; 12:58–67 22158022 10.1038/nrc3171

[3] Obeid M Tesniere A Ghiringhelli F Fimia GM Apetoh L Perfettini JL Calreticulin exposure dictates the immunogenicity of cancer cell death. Nat Med. 2007; 13:54–61 17187072 10.1038/nm1523

[4] Majeti R Chao MP Alizadeh AA Pang WW Jaiswal S Gibbs KD Jr CD47 is an adverse prognostic factor and therapeutic antibody target on human acute myeloid leukemia stem cells. Cell. 2009; 138:286–299 19632179 10.1016/j.cell.2009.05.045 PMC2726837

[5] Willingham SB Volkmer JP Gentles AJ Sahoo D Dalerba P Mitra SS The CD47-signal regulatory protein alpha (sirpa) interaction is a therapeutic target for human solid tumors. Proc Natl Acad Sci U S A. 2012; 109:6662–6667 22451913 10.1073/pnas.1121623109 PMC3340046

[6] Chao MP Alizadeh AA Tang C Myklebust JH Varghese B Gill S Anti-CD47 antibody synergizes with rituximab to promote phagocytosis and eradicate non-hodgkin lymphoma. Cell. 2010; 142:699–713 20813259 10.1016/j.cell.2010.07.044 PMC2943345

[7] Matlung HL Szilagyi K Barclay NA van den Berg TK The CD47-sirpα signaling axis as an innate immune checkpoint in cancer. Immunol Rev. 2017; 276:145–164 28258703 10.1111/imr.12527

[8] Liu X Pu Y Cron K Deng L Kline J Frazier WA CD47 blockade triggers T cell-mediated destruction of immunogenic tumors. Nat Med. 2015; 21:1209–1215 26322579 10.1038/nm.3931 PMC4598283

[9] Tseng D Volkmer JP Willingham SB Contreras-Trujillo H Fathman JW Fernhoff NB Anti-CD47 antibody-mediated phagocytosis of cancer by macrophages primes an effective antitumor T-cell response. Proc Natl Acad Sci U S A. 2013; 110:11103–11108 23690610 10.1073/pnas.1305569110 PMC3703977

[10] Weiskopf K Cancer immunotherapy targeting the CD47/sirpα axis. Eur J Cancer. 2017; 76:100–109 28286286 10.1016/j.ejca.2017.02.013

[11] Melanoma Treatment (PDQ)–Health Professional Version. 2017 National Cancer Institute Available at: *https://www.cancer.gov/types/skin/hp/melanoma-treatment-pdq*. [Accessed 20 March 2019]

[12] Hodi FS Chesney J Pavlick AC Robert C Grossmann KF McDermott DF Combined nivolumab and ipilimumab versus ipilimumab alone in patients with advanced melanoma: 2-year overall survival outcomes in a multicentre, randomised, controlled, phase 2 trial. Lancet Oncol. 2016; 17:1558–1568 27622997 10.1016/S1470-2045(16)30366-7 PMC5630525

[13] Weber JS Hodi FS Wolchok JD Topalian SL Schadendorf D Larkin J Safety profile of nivolumab monotherapy: a pooled analysis of patients with advanced melanoma. J Clin Oncol. 2017; 35:785–792 28068177 10.1200/JCO.2015.66.1389

[14] Beaumont KA Mohana-Kumaran N Haass NK Modeling melanoma *in vitro* and in vivo. Healthcare (Basel). 2013; 2:27–46 27429258 10.3390/healthcare2010027 PMC4934492

[15] Sharma A Kuzu OF Nguyen FD Noory MA Current state of animal (mouse) modeling in melanoma research. Cancer Growth Metastasis. 2015; 8 Suppl 1 81–94 26483610 10.4137/CGM.S21214 PMC4597587

[16] van der Weyden L Patton EE Wood GA Foote AK Brenn T Arends MJ Adams DJ Cross-species models of human melanoma. J Pathol. 2016; 238:152–165 26354726 10.1002/path.4632 PMC4832391

[17] Nishiya A Massoco C Felizzola C Perlmann E Batschinski K Tedardi M Comparative aspects of canine melanoma. Vet Sci. 2016; 3:7 10.3390/vetsci3010007 PMC5644618 29056717

[18] Kedl RM Jordan M Potter T Kappler J Marrack P Dow S CD40 stimulation accelerates deletion of tumor-specific CD8(+) T cells in the absence of tumor-antigen vaccination. Proc Natl Acad Sci U S A. 2001; 98:10811–10816 11526222 10.1073/pnas.191371898 PMC58556

[19] Ritt MG Wojcieszyn J Modiano JF Functional loss of p21/waf-1 in a case of benign canine multicentric melanoma. Vet Pathol. 1998; 35:94–101 9539362 10.1177/030098589803500202

[20] Modiano JF Kokai Y Weiner DB Pykett MJ Nowell PC Lyttle CR Progesterone augments proliferation induced by epidermal growth factor in a feline mammary adenocarcinoma cell line. J Cell Biochem. 1991; 45:196–206 2055947 10.1002/jcb.240450211

[21] Thayanithy V Park C Sarver AL Kartha RV Korpela DM Graef AJ Combinatorial treatment of DNA and chromatin-modifying drugs cause cell death in human and canine osteosarcoma cell lines. PLoS One. 2012; 7:e43720 22957032 10.1371/journal.pone.0043720 PMC3434163

[22] Rütgen BC Hammer SE Gerner W Christian M de Arespacochaga AG Willmann M Establishment and characterization of a novel canine B-cell line derived from a spontaneously occurring diffuse large cell lymphoma. Leuk Res. 2010; 34:932–938 20153049 10.1016/j.leukres.2010.01.021

[23] Weiskopf K Ring AM Ho CC Volkmer JP Levin AM Volkmer AK Engineered sirpα variants as immunotherapeutic adjuvants to anticancer antibodies. Science. 2013; 341:88–91 23722425 10.1126/science.1238856 PMC3810306

[24] Liu J Wang L Zhao F Tseng S Narayanan C Shura L Pre-clinical development of a humanized anti-CD47 antibody with anti-cancer therapeutic potential. PLoS One. 2015; 10:e0137345 26390038 10.1371/journal.pone.0137345 PMC4577081

[25] Curtsinger JM Lins DC Mescher MF Signal 3 determines tolerance versus full activation of naive CD8 T cells: dissociating proliferation and development of effector function. J Exp Med. 2003; 197:1141–1151 12732656 10.1084/jem.20021910 PMC2193970

[26] Gerner MY Casey KA Mescher MF Defective MHC class II presentation by dendritic cells limits CD4 T cell help for antitumor CD8 T cell responses. J Immunol. 2008; 181:155–164 18566380 10.4049/jimmunol.181.1.155 PMC2587216

[27] Gerner MY Mescher MF Antigen processing and MHC-II presentation by dermal and tumor-infiltrating dendritic cells. J Immunol. 2009; 182:2726–2737 19234167 10.4049/jimmunol.0803479 PMC2712950

[28] Weiskopf K Anderson KL Ito D Schnorr PJ Tomiyasu H Ring AM Eradication of canine diffuse large B-cell lymphoma in a murine xenograft model with CD47 blockade and anti-CD20. Cancer Immunol Res. 2016; 4:1072–1087 27856424 10.1158/2326-6066.CIR-16-0105 PMC5454476

[29] Ngo M Han A Lakatos A Sahoo D Hachey SJ Weiskopf K Antibody therapy targeting CD47 and CD271 effectively suppresses melanoma metastasis in patient-derived xenografts. Cell Rep. 2016; 16:1701–1716 27477289 10.1016/j.celrep.2016.07.004

[30] Sockolosky JT Dougan M Ingram JR Ho CC Kauke MJ Almo SC Durable antitumor responses to CD47 blockade require adaptive immune stimulation. Proc Natl Acad Sci U S A. 2016; 113:E2646–E2654 27091975 10.1073/pnas.1604268113 PMC4868409

[31] Barkal AA Weiskopf K Kao KS Gordon SR Rosental B Yiu YY Engagement of MHC class I by the inhibitory receptor LILRB1 suppresses macrophages and is a target of cancer immunotherapy. Nat Immunol. 2018; 19:76–84 29180808 10.1038/s41590-017-0004-z PMC5832354

[32] Furness AJ Vargas FA Peggs KS Quezada SA Impact of tumour microenvironment and fc receptors on the activity of immunomodulatory antibodies. Trends Immunol. 2014; 35:290–298 24953012 10.1016/j.it.2014.05.002

[33] Kontermann RE Brinkmann U Bispecific antibodies. Drug Discov Today. 2015; 20:838–847 25728220 10.1016/j.drudis.2015.02.008

