# Supplementary material for: Evolutionarily conserved resistance to phagocytosis observed in melanoma cells is insensitive to upregulation of pro-phagocytic signals and to CD47 blockade
Source: Melanoma Res. 2019 Jun 12;30(2):147–58. doi: 10.1097/CMR.0000000000000629 (PMC6906263; doi:10.1097/CMR.0000000000000629)
Supplement: Supplementary file 1 [file mr-30-147-s001.pdf]

Supplemental Digital Content 1: Supplemental Methods

Macrophage derivation: Mouse and human macrophages were derived as previously described (5, 23). Briefly, murine bone marrow cells were isolated from C57BL/6 and Nod.Cg-Prkdc^{scid} IL2rg^{tm1Wjl}/SzJ (NSG) mice, then cultured in IMDM+Glutamax supplemented with 10% fetal bovine serum, 100 U/mL penicillin and 100 μg/mL streptomycin, and 10 ng/mL murine M-CSF for 7 days. Human macrophages were derived from peripheral blood samples (Stanford Blood Center) that underwent density gradient centrifugation with Ficoll-Plaque, followed by monocyte-isolation using anti-CD14 MicroBeads (Miltenyi Biotech), and differentiation into macrophages by culturing for 7 days in IMDM+GlutaMax supplemented with 10% AB-Human Serum, 100 U/mL penicillin and 100 μg/mL streptomycin.

Clustering of TCGA expression data for extracellularly expressed proteins: The results published here are in part based upon data generated by the TCGA Research Network: http://cancergenome.nih.gov/. Uniformly processed TCGA expression data in transcripts per million (TPM) were obtained from GEO accession GSE62944 (26). Extracellular genes were identified using the COMPARTMENTS database (27) and GOType labels plasma membrane, cell surface, extracellular region, extracellular matrix and a score > 4. Expression data for these extracellular genes (n = 5,082) in the cancer types of interest (DLBC, COAD, BRCA, LUAD, LUSC and SKCM) was isolated and used in further analysis. Variance of expression was calculated for each gene across all tumor samples and the top 500 most variable genes were clustered based on gene expression values using Euclidean distance.

siRNA literature-based selection: 48 genes were included in the siRNA panel (Table, Supplemental Digital Content 7A). All of these genes (except Bcl-2) are expressed on the surface of melanoma cells based on a literature search (PubMed) and using the COMPARTMENTS online subcellular localization database (27). Bcl-2 was chosen as a control on the basis that knockdown should increase apoptosis. The other genes were chosen based on 1 of 4 criteria: (1) the gene had been identified as a “don’t eat me” signal, (2) gene expression was restricted to melanoma cells, (3) the gene had been shown to contribute to melanoma cell growth and metastasis, and/or (4) the gene had been associated with immune suppression in the tumor microenvironment.

siRNA Mediated Knockdown: ON-TARGETplus siRNA pools targeting 48 melanoma proteins, siRNAs targeting CD47, the non-targeting control siRNA pool, and the siGLO Red Transfection Indicator were validated by and purchased from Dharmacon. Utilizing a random number generator, we combined the 48 siRNAs into pools of three siRNAs plus a siRNA targeting CD47 (Table, Supplemental Digital Content 7B). Each siRNA was used in four different pools. siRNA pools were tested in seven groups, each with its own set of controls (untransfected M14 cells, M14 cells transfected with non-targeting siRNA, and Raji lymphoma cells as a positive control for phagocytosis). 230,000 M14-GFP cells were plated 24 hours prior to transfection in antibiotic-free DMEM medium. Cells were transfected using TransIT-TKO reagent (Mirus Bio LLC) according to the manufacturer’s protocol with either 100 μM of each siRNA in the pool or 400 μM non-targeting control siRNA. Cells were cultured for 48 hours prior to analysis or placement in phagocytosis assays.

RNA preparation and quantitative reverse-transcriptase PCR (qRT-PCR): RNA was

isolated and purified from cells using the RNeasy Mini Kit (Qiagen) and concentrations were measured using a NanoDrop ND-1000 UV–vis spectrophotometer. cDNA was synthesized using the QuantiTect Reverse Transcription Kit (Qiagen) according to the manufacturer's protocol. Eight genes targeted in the siRNA pool were chosen by a random number generator and checked to ensure that multiple protein families were represented by these genes. Primers were designed using NCBI's Primer Blast tool (see table below). qRT-PCR was performed using a LightCycler® 96 with FastStart SYBR Green Master Mix (Roche Applied Science). Samples in which the template was omitted were used as negative controls. Relative mRNA values were expressed as delta-Ct values normalized to GAPDH.

Primer Sequences for qRT-PCR Analysis

Gene ID	Forward Primer (5'-3')	Reverse Primer (5'-3')
CD47	CAACCTCCTAGGAATAACTGAAGTG	GGGTCTCATAGGTGACAACCAG
CD81	AATTTCGTCTTCTGGCTGGCT	CCAACGAACATCATGACAGCG
MCAM	TGGGCGCTGTCCTCTATTTC	GTTCGCTCTTACGAGACGGG
JAM3	GACAAGTGACCCCAGGATCG	ACCTCACAGCGATAAAGGGC
ICAM1	TGACCGTGAATGTGCTCTCC	TCCCTTTTTGGGCCTGTTGT
LGALS1	CCTGACGCTAAGAGCTTCGT	GGAAGGGAAAGACAGCCTCC
MLANA	TGTGCCCTGACCCTACAAGA	AATACCAACAGCCGATGAGCA

CD47 knockout in M14 and Raji cells by CRISPR/Cas9 editing:

Generation of Customized Guide RNA Expression Construct: Gene editing experiments were done by the Masonic Cancer Center, University of Minnesota Gene Editing Shared Resource. To knockout CD47 expression, a sense stranded (S1) sgRNA targeting exon 2 of CD47 was cloned into a CRISPR/Cas9-GFP expression vector (PX458). Briefly, a sense oligo 5'-CACCGATCGAGCTAAAATATCGTGT-3' and an anti-sense oligo 5'-AAACACACGATATTTTAGCTCGATC-3', containing four base-pair

overhangs compatible with PX458 BbsI restriction enzyme digestion sites, were annealed and ligated into PX458. Correct incorporation of this sgRNA sequence was confirmed by Sanger sequencing (Genewiz, South Plainfield, NJ, USA) using a vector specific primer located 5' to the U6 promoter 5'-GGCCTTTTGCTGGCCTTTTGCTC-3'.

M14 and Raji Transfections and screening for CD47 knockout clones: The PX458-CD47-exon 2 S1 sgRNA plasmid was electroporated into M14 or Raji cells using a Neon electroporator (Invitrogen) and allowed to recover for two days. GFP positive cells were then collected by FACS sorting. The efficiency of indel formation was determined by Tracking of Indels by Decomposition (TIDE) analysis (tide.deskgen.com). Briefly, genomic DNA was collected from the polyclonal targeted population and PCR was performed using primers that span exon 2 of CD47, CD47 exon 2 ScrF1 5'-AGTGAACAATGGAAATGTTGCTGT-3' and CD47 exon 2 ScrR1 5'-CCCAGGACCAATCAGCCAAA-3' to produce a 756 bp amplicon. Amplicons generated from either non-targeted (reference control) or the CRISPR/Cas9 targeted population were submitted for Sanger sequencing. Upon the determination of efficient Cas9 cutting (17.6%), the polyclonal population was sub-cloned by limited dilution into 96-well plates. Single cell clones were identified and expanded further into 24-well plates for genomic DNA collection and PCR screening. Resulting amplicons from individual clones were further cloned into pCR4-TOPO vector using a TOPO TA cloning kit (Invitrogen). Plasmid DNA was isolated from individual transformants and sequenced by Sanger sequencing using the CD47 exon 2 ScrF1 primer.

Upon screening for indels, it was determined that M14 cells are triploid for chromosome 3 where the CD47 gene resides. One clone (clone 9), was identified as containing

indels on at least two alleles that would put the CD47 mRNA out of frame starting in exon 2 of the transcript (1 bp T insertion and 2 bp deletion) as well as one that deletes the exon 2 splice donor (15 bp deletion). Two clones (clone 8 and clone 17) recovered from the screened clones with no apparent Cas9 editing were used as WT CD47 controls.

For Raji cells, the indel frequency was determined to be 48.3%. Sequence analysis of a total of 13 clones revealed two clones (clone 1 and clone 13) containing homozygous deletions within exon 2. Clone 1 contained a homozygous 188 bp deletion within exon 2 that would put the CD47 mRNA out of frame while clone 13 contained a 116 bp deletion that removed exon 2's splice donor. One clone (clone 17) containing a biallelic 3 bp in-frame deletion was used as a pseudo WT control as it still expressed CD47, although it was missing R132.

Reverse Phase Protein Array (RPPA): Parental Raji lymphoma and M14 melanoma cells, CD47 CRISPR knockout cells, and the CRISPR controls were cultured in the presence of no antibody, huIgG4, or hu5F9-G4 for 2 hours, harvested, and submitted to the Core Facility at MD Anderson Cancer Center for RPPA analysis. Samples were prepared and analyzed using 297 unique antibodies as described (26). RPPA slide densities were quantified using Array-Pro Analyzer 6.3, and relative protein levels for each sample were determined using SuperCurve_1.5.0 via SuperCurveGUI_2.1.1 (27). All relative protein level data points were normalized for protein loading and transformed to linear values. RPPA slide quality was assessed as previously described (28), and only slides with a QC score above 0.8 (range: 0-1) were utilized in the analysis. Initial PCA of normalized protein data for each cell type identified two outlier samples that

were removed from further analysis (M14 sample 4A-1 and Raji sample 2B-1). The remaining samples were corrected for batch effects based on the day of cell collection using the RemoveBatchEffects from the limma package in R. Batch corrected data was used as the input for clustering and heatmap generation. Complete hierarchical clustering using Euclidean distance was applied to scaled protein data by the pheatmap command in R to cluster samples and proteins by their expression patterns.

CD47 Restoration: A CD47 expression plasmid (Human CD47 cDNA ORF Clone GFPSpark Tag) was obtained from Sino Biological and transfected into the CD47 CRISPR knockout Raji lymphoma cells as well as CRISPR control Raji cells (Neon Transfection System) according to the manufacturer’s protocol using 1350 volts for 30 msec (1 pulse).
