# Supplementary material for: Evolutionarily conserved resistance to phagocytosis observed in melanoma cells is insensitive to upregulation of pro-phagocytic signals and to CD47 blockade
Source: Melanoma Res. 2019 Jun 12;30(2):147–58. doi: 10.1097/CMR.0000000000000629 (PMC6906263; doi:10.1097/CMR.0000000000000629)
Supplement: Supplementary file 2 [file mr-30-147-s002.pdf]

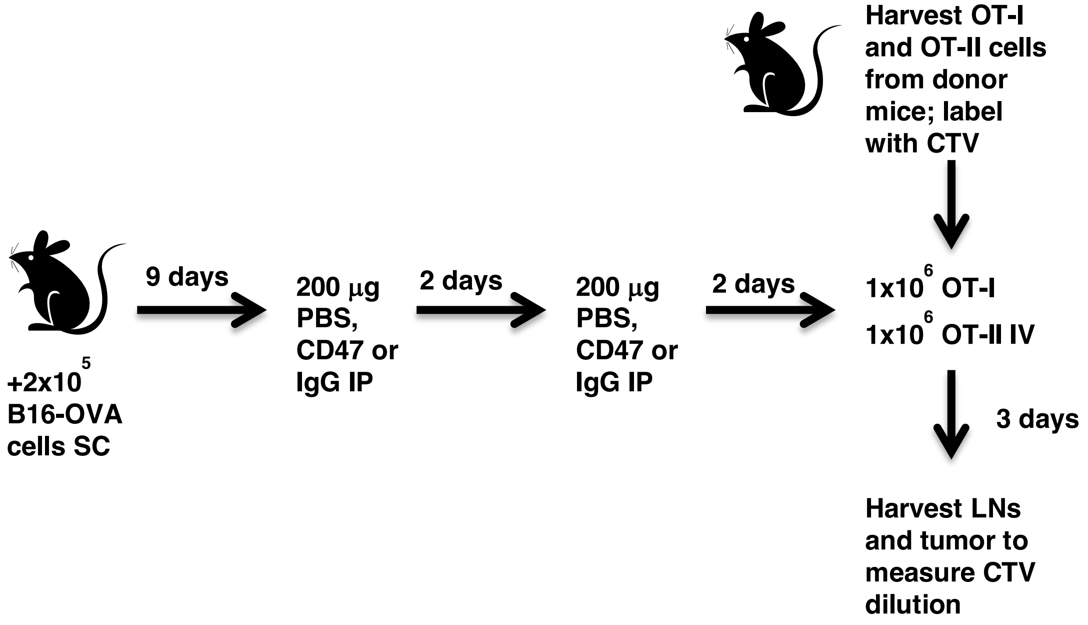

Supplemental Digital Content 2: Schematic of the experimental design to assess the effects of CD47 blockade on antigen-specific T cell activation *in vivo*
