# Supplementary material for: Evolutionarily conserved resistance to phagocytosis observed in melanoma cells is insensitive to upregulation of pro-phagocytic signals and to CD47 blockade
Source: Melanoma Res. 2019 Jun 12;30(2):147–58. doi: 10.1097/CMR.0000000000000629 (PMC6906263; doi:10.1097/CMR.0000000000000629)
Supplement: Supplementary file 3 [file mr-30-147-s003.pdf]

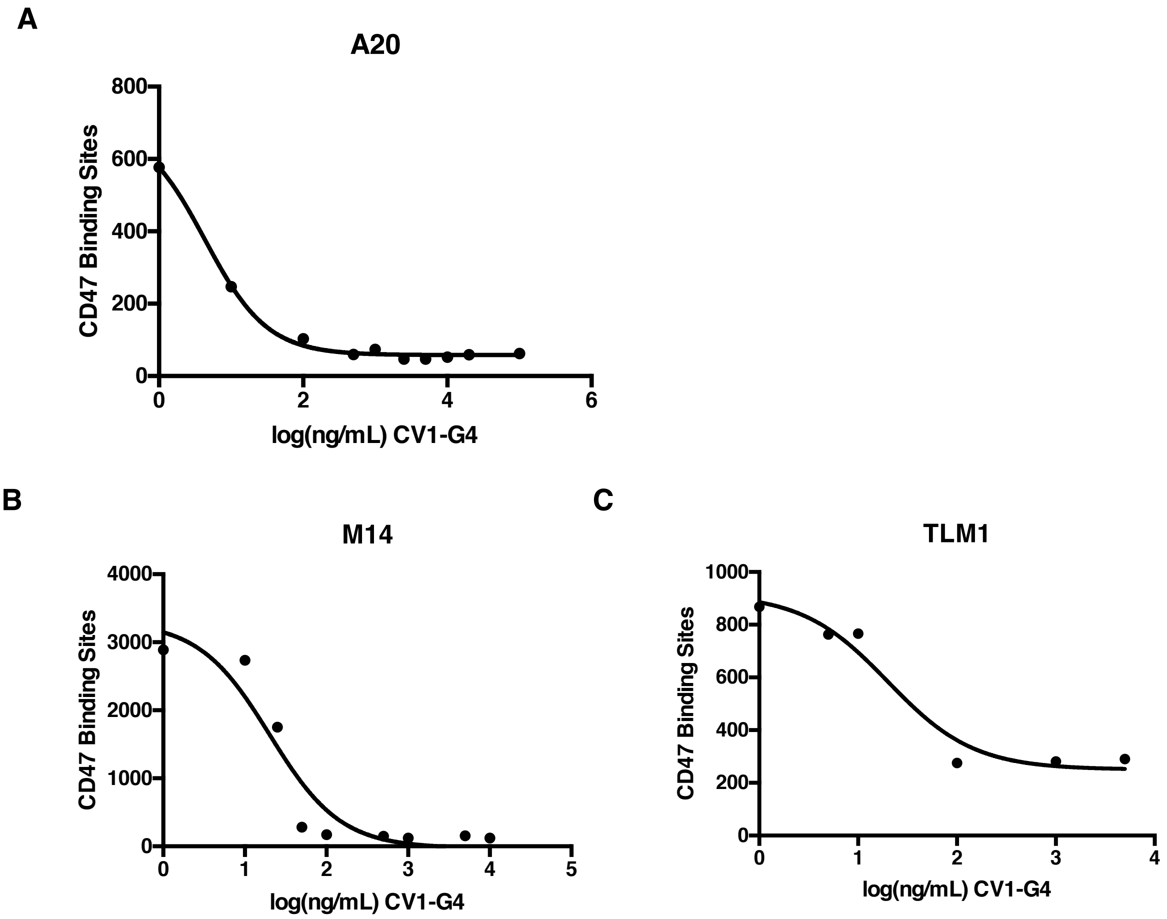

Supplemental Digital Content 3: Efficacy of CV1-G4 Blockade. Mouse A20 lymphoma cells (A), human M14 melanoma cells (B), or canine TLM1 melanoma cells (C) were incubated with unlabeled CV1-G4 at the indicated concentrations for 15 minutes, followed by subsequent labeling with Alexa-488 hu5F9 mAb (human and canine cells) or PE-MIAP301 (mouse cells). The ability of the labeled antibody to bind CD47 on the cell surface was evaluated by flow cytometry (MFI).
