# Supplementary material for: Evolutionarily conserved resistance to phagocytosis observed in melanoma cells is insensitive to upregulation of pro-phagocytic signals and to CD47 blockade
Source: Melanoma Res. 2019 Jun 12;30(2):147–58. doi: 10.1097/CMR.0000000000000629 (PMC6906263; doi:10.1097/CMR.0000000000000629)
Supplement: Supplementary file 4 [file mr-30-147-s004.pdf]

A

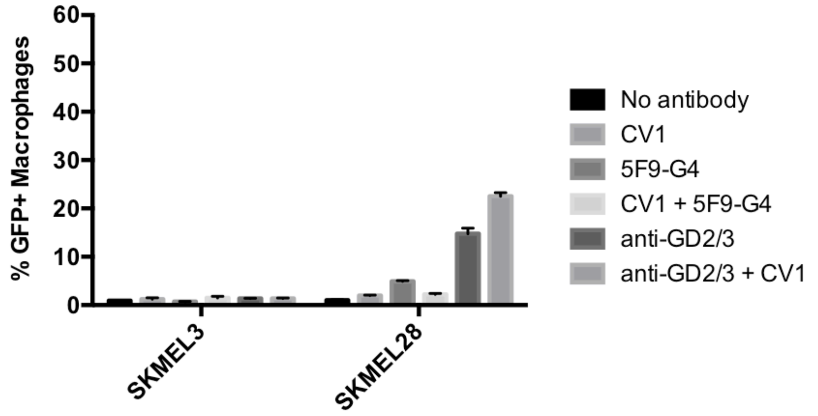


B

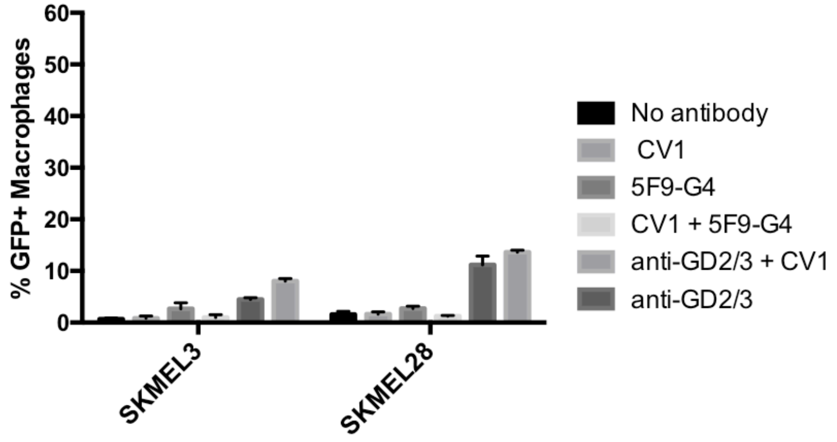


**Supplemental Digital Content 4: Human melanoma cell lines display resistance to CD47 blockade.** A. Phagocytosis of human SKMEL3 and SKMEL28 cell lines by mouse NSG macrophages. Tumor cells were incubated with bone-marrow derived NSG macrophages in the presence of control IgG4, 5F9-G4, and/or anti-GD2/3 antibodies. Phagocytosis was quantified as the percent of F4/80$^+$ J774 cells that engulfed CFSE$^+$ tumor cells per total F4/80$^+$ population. The data are a summary of one experiment repeated in triplicate (mean± SEM). B. Phagocytosis of human SKMEL3 and SKMEL28 cell lines by human macrophages. Tumor cells were incubated with primary human macrophages in the presence of control IgG4, 5F9-G4, and/or anti-GD2/3 antibodies, and phagocytosis was quantified as in A. For SKMEL3, the data represent 2 experiments (using 2 different human donors) repeated in triplicate (mean± SEM). For SKMEL28, the data represent 4 experiments (using 4 different human donors) repeated in triplicate (mean± SEM).
