# Supplementary material for: Evolutionarily conserved resistance to phagocytosis observed in melanoma cells is insensitive to upregulation of pro-phagocytic signals and to CD47 blockade
Source: Melanoma Res. 2019 Jun 12;30(2):147–58. doi: 10.1097/CMR.0000000000000629 (PMC6906263; doi:10.1097/CMR.0000000000000629)
Supplement: Supplementary file 5 [file mr-30-147-s005.pdf]

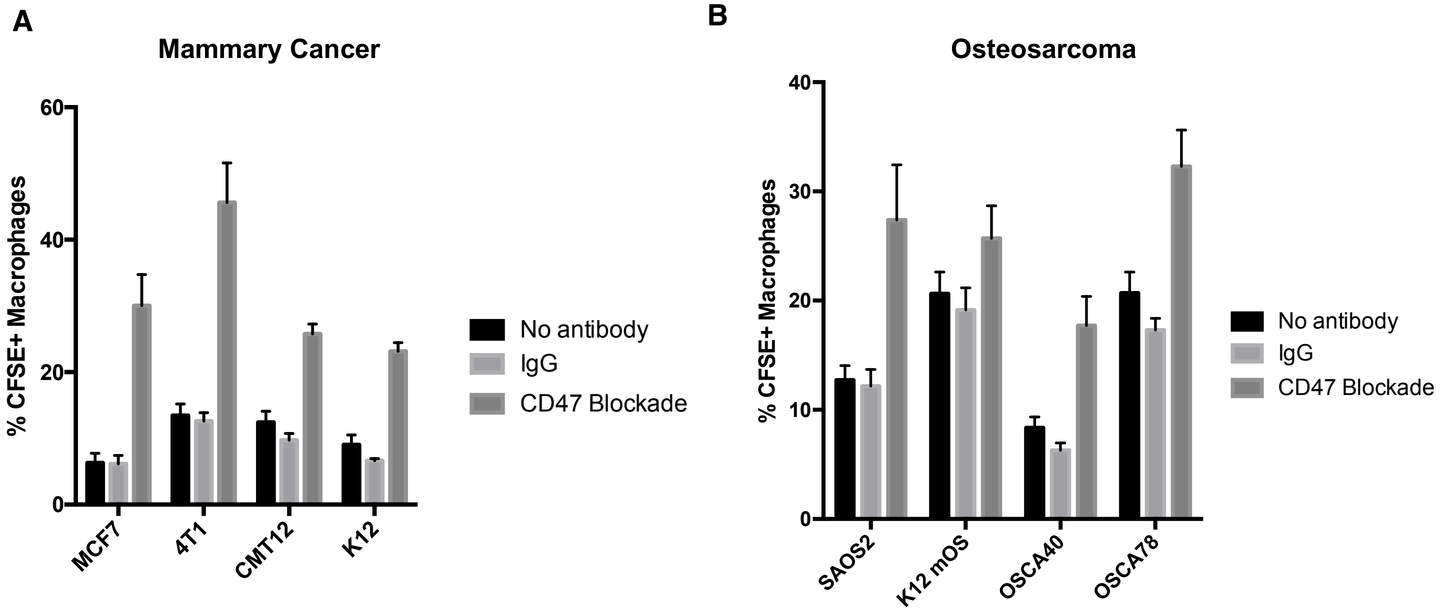

Supplemental Digital Content 5: CD47 Blockade Enhances Phagocytosis of Mammary Cancer and Osteosarcoma Cell Lines. CFSE-labeled tumor cells were incubated with J774 macrophages in the presence of control IgG4 or CV1-G4. Phagocytosis was quantified as the percent of F4/80$^+$ J774 cells that engulfed CFSE$^+$ tumor cells per total F4/80$^+$ population. The data are a summary of two experiments were performed in triplicate (mean ± SEM). A. Phagocytosis of human (MCF7), mouse (4T1), canine (CMT12), and feline (K12) mammary cancer cells. B. Phagocytosis of human (SAOS2), mouse (K12 mOS), and canine (OSCA40 and OSCA78) osteosarcoma cells.
