# Supplementary material for: Evolutionarily conserved resistance to phagocytosis observed in melanoma cells is insensitive to upregulation of pro-phagocytic signals and to CD47 blockade
Source: Melanoma Res. 2019 Jun 12;30(2):147–58. doi: 10.1097/CMR.0000000000000629 (PMC6906263; doi:10.1097/CMR.0000000000000629)
Supplement: Supplementary file 6 [file mr-30-147-s006.pdf]

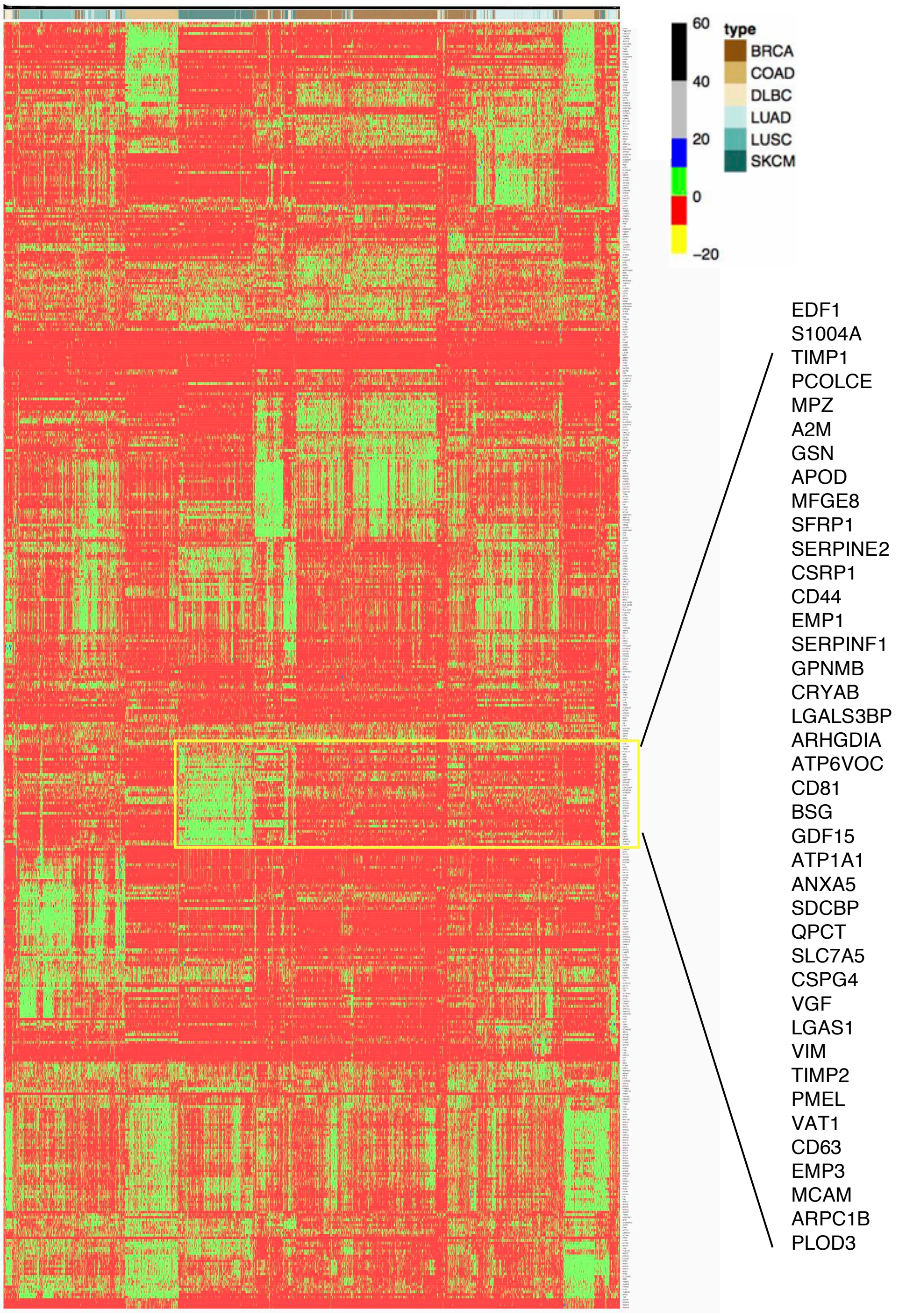

Supplemental Digital Content 6: Identification of genes expressed on the plasma membrane of melanoma cells. Heatmap of the top 500 most variable extracellular genes from a subset of TCGA cancer types. Clustering was performed based on gene expression in transcripts per million (by rows). Gene expression values are centered and scaled.
