# Supplementary material for: Evolutionarily conserved resistance to phagocytosis observed in melanoma cells is insensitive to upregulation of pro-phagocytic signals and to CD47 blockade
Source: Melanoma Res. 2019 Jun 12;30(2):147–58. doi: 10.1097/CMR.0000000000000629 (PMC6906263; doi:10.1097/CMR.0000000000000629)
Supplement: Supplementary file 7 [file mr-30-147-s007.pdf]

Supplemental Digital Content 7A: Table displaying the targets of the cell surface siRNA panel

Assigned Number	Gene Symbol	Assigned Number	Gene Symbol
1	BCL2	25	JAM3
2	CD200	26	ITGAV
3	PECAM1	27	ITGA2
4	CD151	28	ITGA3
5	CD81	29	ITGA4
6	SERPINE1	30	ITGA5
7	ANXA1	31	ITGA6
8	MIF	32	ITGA7
9	SELPLG	33	ITGB1
10	ECM1	34	ITGB3
11	CDH1	35	ITGB4
12	CDH2	36	ITGB5
13	CDH3	37	ICAM1
14	CLDN1	38	AGER
15	CLDN12	39	LGALS1
16	ALCAM	40	LGALS3
17	MCAM	41	DSC3
18	NCAM1	42	CAV1
19	CEACAM1	43	ST8SIA1
20	BCAM	44	MLANA
21	L1CAM	45	PMEL
22	MADCAM1	46	TRPC1
23	EPCAM	47	MFGE8
24	F11R	48	PXN

Supplemental Digital Content 7B: Table displaying the siRNA Knockdown Pools

Group 1		Group 2		Group 3		Group 4	
**Pool**	**siRNA Numbers**	**Pool**	**siRNA Numbers**	**Pool**	**siRNA Numbers**	**Pool**	**siRNA Numbers**
Pool 1	34, 5, 42	Pool 1	12, 37, 44	Pool 1	26, 35, 48	Pool 1	1, 45, 19
Pool 2	21, 38, 20	Pool 2	43, 4, 21	Pool 2	14, 41, 29	Pool 2	34, 30, 46
Pool 3	13, 31, 2	Pool 3	8, 35, 18	Pool 3	20, 28, 44	Pool 3	37, 24, 18
Pool 4	24, 6, 36	Pool 4	23, 11, 41	Pool 4	27, 25, 5	Pool 4	39, 32, 33
Pool 5	30, 22, 33	Pool 5	7, 24, 48	Pool 5	11, 37, 9	Pool 5	36, 31, 2
Pool 6	43, 27, 15	Pool 6	26, 31, 14	Pool 6	3, 31, 24	Pool 6	9, 43, 44
Pool 7	48, 12, 40	Pool 7	28, 29, 6	Pool 7	46, 2, 13	Pool 7	28, 22, 17
Pool 8	23, 45, 4	Pool 8	3, 22, 19	Pool 8	4, 34, 33	Pool 8	23, 16, 6
Pool 9	37, 25, 39	Pool 9	20, 47, 15	Pool 9	10, 47, 45	Pool 9	48, 48, 13
Pool 10	28, 9, 11	Pool 10	25, 30, 13	Pool 10	15, 19, 36	Pool 10	42, 40, 27
Pool 11	19, 3, 1	Pool 11	42, 5, 38	Pool 11	16, 42, 23	Pool 11	3, 11, 29
Pool 12	14, 16, 32	Pool 12	17, 34, 40	Pool 12	7, 21, 12	Pool 12	47, 10, 8
Pool 13	8, 18, 41	Pool 13	36, 46, 32	Pool 13	18, 43, 1	Pool 13	15, 26, 25
Pool 14	39, 47, 35	Pool 14	45, 10, 1	Pool 14	39, 30, 17	Pool 14	12, 7, 4
Pool 15	7, 17, 46	Pool 15	39, 9, 2	Pool 15	22, 40, 8	Pool 15	5, 41, 21
Pool 16	10, 26, 44	Pool 16	33, 16, 27	Pool 16	32, 6, 38	Pool 16	14, 20, 35

Supplemental Digital Content 7C: Table displaying the repeated siRNA knockdown pools

Group Number	Genes Targeted	Assigned Numbers
Group 1 Pool 1	ITGB3, CD81, CAV1	34, 5, 42
Group 1 Pool 5	ITGA5, MADCAM1, ITGB1	30, 22, 33
Group 1 Pool 10	ITGA3, SELPG, CDH1	28, 9, 11
Group 2 Pool 9	BCAM, MFGE8, CLDN12	20, 47, 15
Group 2 Pool 12	MCAM, ITGB3, LGALS1	17, 34, 39
