# Supplementary material for: Evolutionarily conserved resistance to phagocytosis observed in melanoma cells is insensitive to upregulation of pro-phagocytic signals and to CD47 blockade
Source: Melanoma Res. 2019 Jun 12;30(2):147–58. doi: 10.1097/CMR.0000000000000629 (PMC6906263; doi:10.1097/CMR.0000000000000629)
Supplement: Supplementary file 8 [file mr-30-147-s008.pdf]

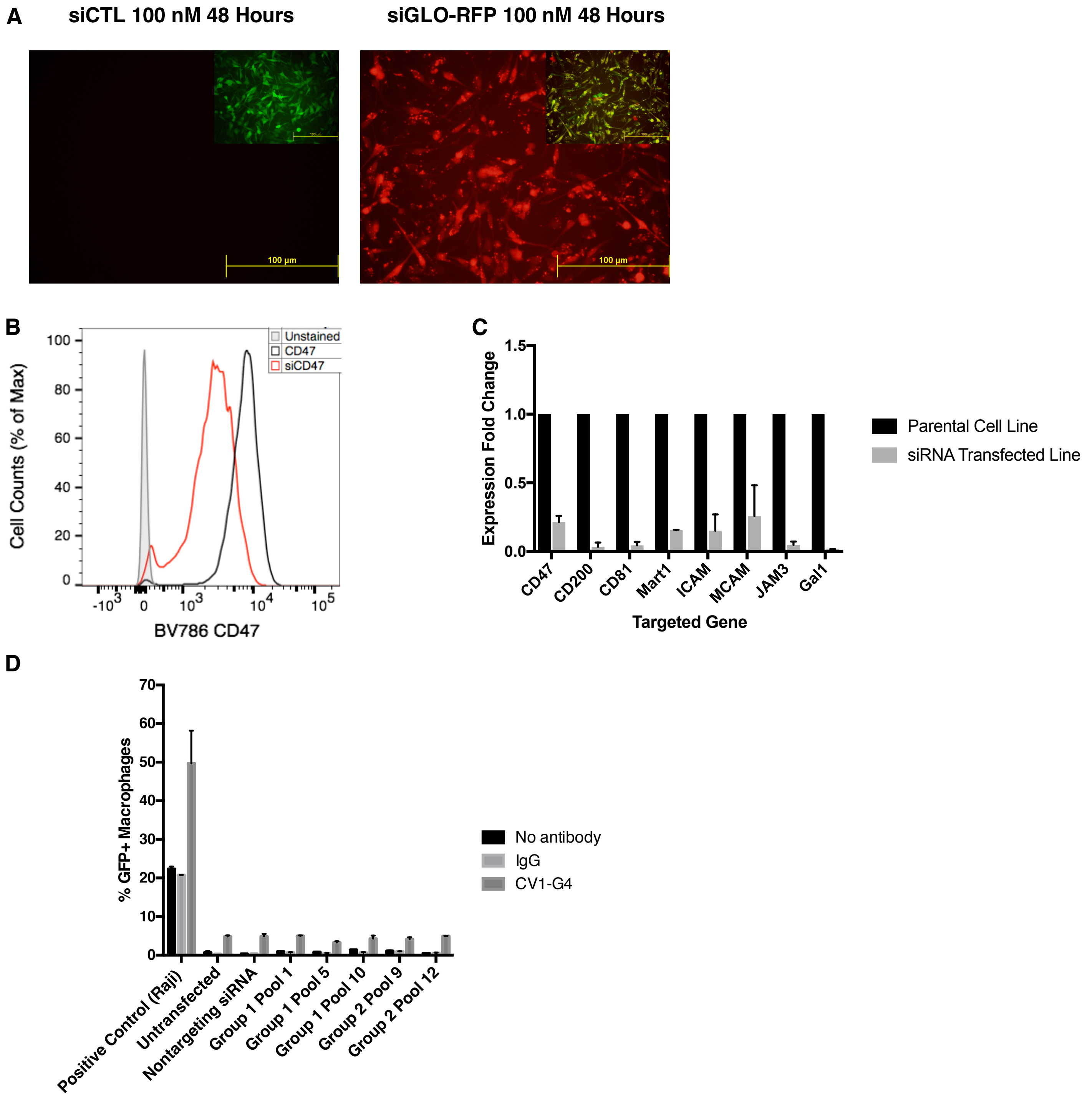

Supplemental Digital Content 8: siRNA mediated knockdown of 48 candidate proteins does not enhance melanoma cell phagocytosis. A. Fluorescent microscopy images of M14-GFP cells 48 hours following transfection with 100 nM non-targeting siRNA (siCTL) or 100 nM siGLO-RFP. B. CD47 expression of parental M14-GFP cells and cells transfected with siCD47 was evaluated by flow cytometry 48 hours after transfection. C. Expression levels of eight target genes were evaluated by quantitative RT-PCR 48 hours after transfection with siRNA. Gene expression was normalized to GAPDH among samples. Bars represent three transfections (mean± SEM). D. 48 hours post-transfection, M14-GFP cells were co-cultured with J774 macrophages in the presence of control IgG4 or CV1-G4. Phagocytosis was quantified as the percent of F4/80$^+$ J774 cells that engulfed CFSE$^+$ tumor cells per total F4/80$^+$ population. Bars represent one experiments were performed in duplicate (mean ± SEM).
