# Supplementary material for: Evolutionarily conserved resistance to phagocytosis observed in melanoma cells is insensitive to upregulation of pro-phagocytic signals and to CD47 blockade
Source: Melanoma Res. 2019 Jun 12;30(2):147–58. doi: 10.1097/CMR.0000000000000629 (PMC6906263; doi:10.1097/CMR.0000000000000629)
Supplement: Supplementary file 9 [file mr-30-147-s009.pdf]

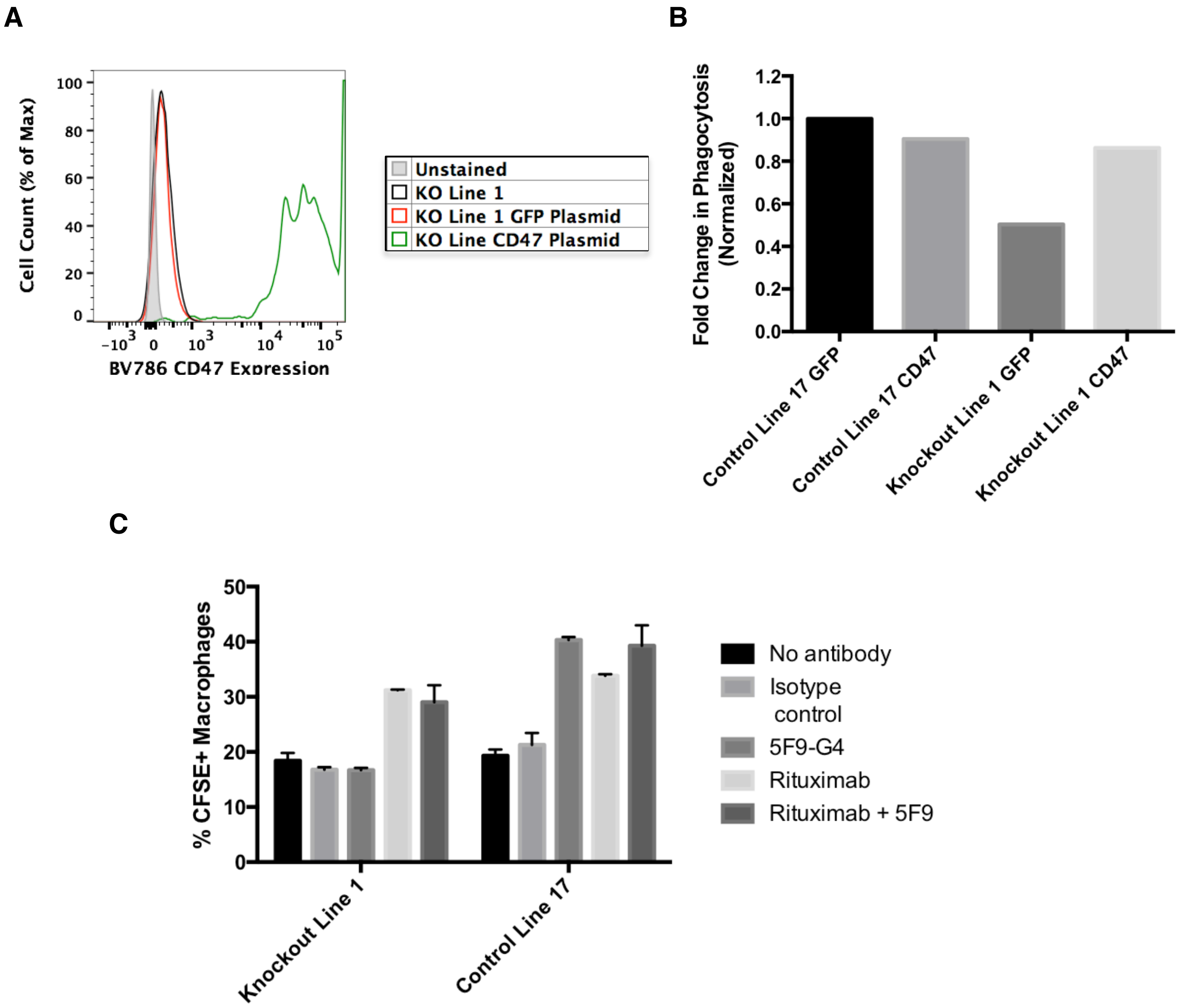

Supplemental Digital Content 9: Restoration of CD47 expression or addition of a tumor specific antibody reestablishes lymphoma cell sensitivity to CD47 blockade. CD47 knockout line 1 and control line 17 were transfected with a GFP control plasmid or a CD47 expression plasmid. A. Re-expression of CD47 in Raji knockout (KO) line 1 was confirmed by flow cytometry using a CD47 antibody conjugated to BV786. B. GFP-labeled tumor cells were incubated with J774 macrophages in the presence of control IgG4 or 5F9-G4. Phagocytosis was quantified as the percent of F4/80+ J774 cells that engulfed GFP+ tumor cells per total F4/80+ population. Fold change was calculated as the change in phagocytosis from cells treated with IgG4 to cells treated with 5F9-G4. Bars represent one experiment. C. Raji CD47 knockout cells and control cells were incubated with J774 macrophages in the presence of control IgG4, 5F9-G4, Rituximab, or a combination of antibodies. Phagocytosis was quantified as the percent of F4/80+ J774 cells that engulfed CFSE+ tumor cells per total F4/80+ population. Bars represent one experiment repeated in duplicate (mean ± SEM).
