# Supplementary material for: Evolutionarily conserved resistance to phagocytosis observed in melanoma cells is insensitive to upregulation of pro-phagocytic signals and to CD47 blockade
Source: Melanoma Res. 2019 Jun 12;30(2):147–58. doi: 10.1097/CMR.0000000000000629 (PMC6906263; doi:10.1097/CMR.0000000000000629)
Supplement: Supplementary file 10 [file mr-30-147-s010.pdf]

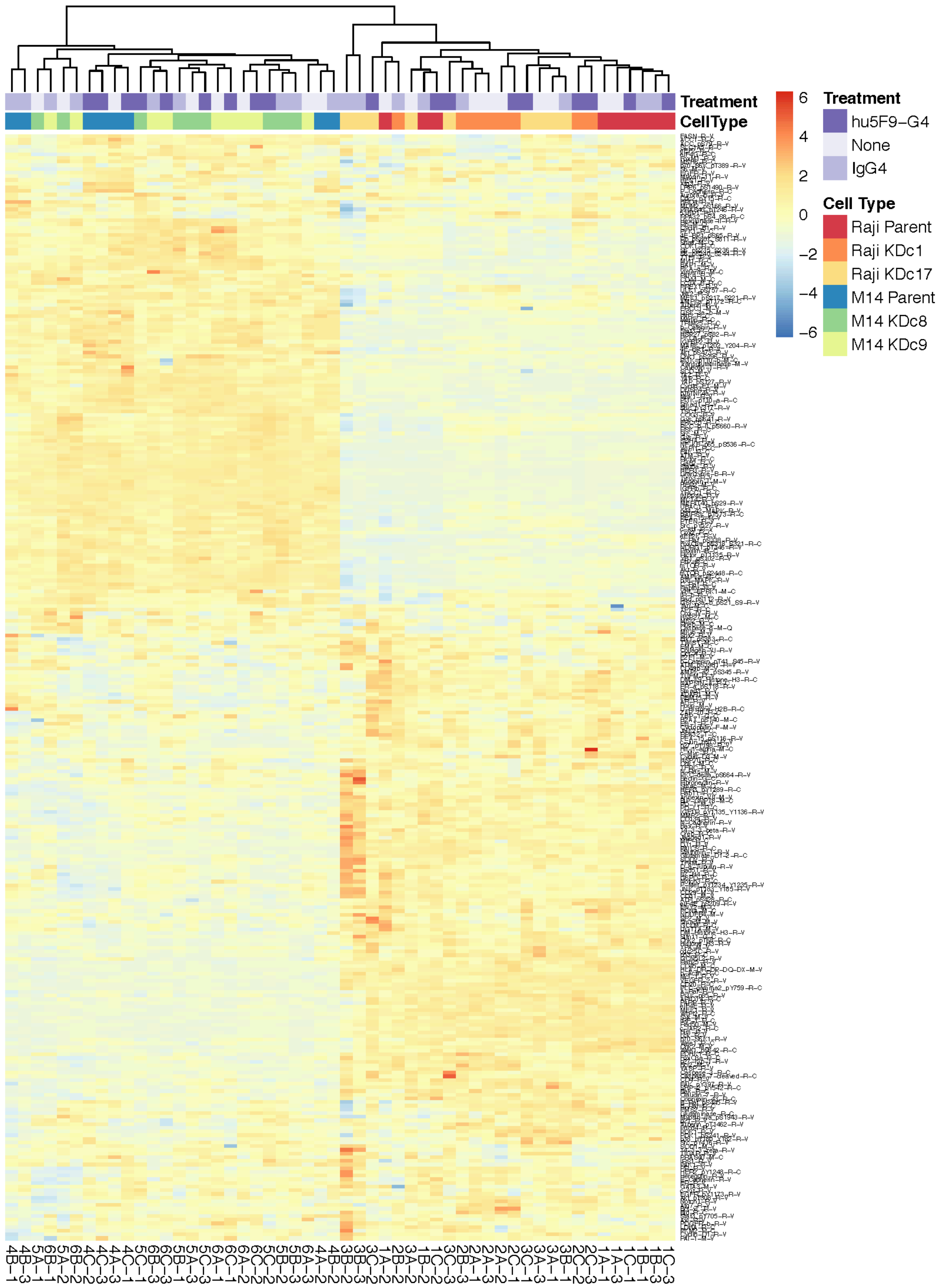

Supplemental Digital Content 10: Proteomics analysis does not reveal differences in cell signaling following CD47 antibody blockade. Raji lymphoma and M14 melanoma parental cells, CD47 knockout cells (Raji KDc1 and M14 KDc9), and CRISPR control cells (Raji KDc17 and M14 KDc8) were incubated with either no antibody, an isotype control, or a CD47 blocking antibody (5F9-G4) for two hours prior to submission for proteomics analysis using a reverse phase protein array (RPPA). Plot shows unsupervised clustering of RPPA data. Sample and treatment are indicated by color at the top of the plot. Protein expression is given as a gradient from high expression (red) to low (blue). Dendrogram indicates euclidean distance between samples.
